# An Ionic Liquid-Based Biorefinery Approach for Duckweed Utilization

**DOI:** 10.1021/acssusresmgt.3c00008

**Published:** 2024-04-30

**Authors:** Anton E. J. Firth, Pedro Y. S. Nakasu, Paul S. Fennell, Jason P. Hallett

**Affiliations:** Department of Chemical Engineering, Imperial College London, London SW7 2AZ, United Kingdom

**Keywords:** metal contamination, pretreatment, duckweed, wastewater remediation, phytoremediation

## Abstract

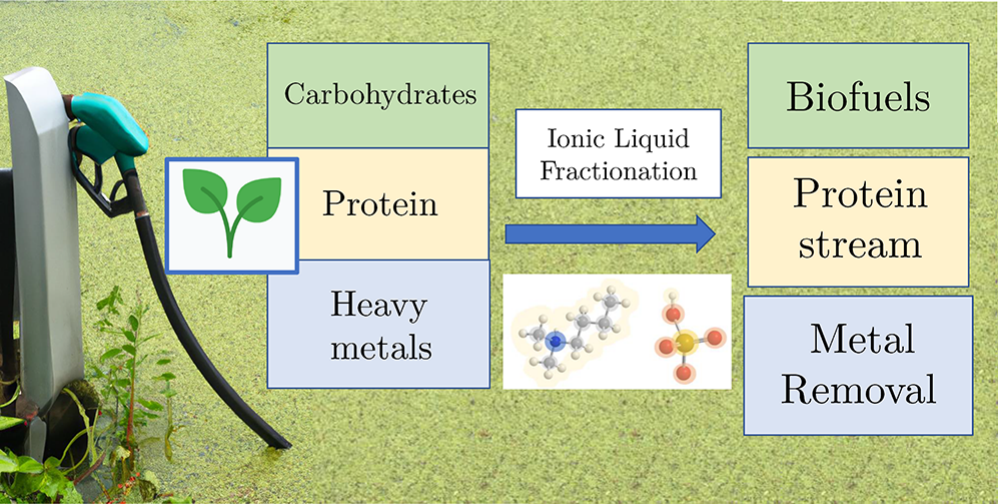

This study establishes a foundation for the ionic liquid (IL) pretreatment of duckweed biomass. An optimized IL-based process was designed to exploit the unique properties of duckweed including efficient metal removal, potential starch accumulation, and protein accumulation. Two ILs, namely, dimethylethanolammonium formate ([DMEtA][HCOO]) and *N,N*-dimethylbutylammonium hydrogen sulfate ([DMBA][HSO_4_]), were investigated for the pretreatment of two duckweed species (*Spirodela polyrhiza* and *Lemna minor*). The evaluation focused on starch recovery, sugar release, protein recovery, and metal extraction capabilities. [DMEtA][HCOO] demonstrated near-quantitative starch recoveries at 120 °C, while [DMBA][HSO_4_] showed similar performance at 90 °C within a reaction time of 2 h. Saccharification yields for most pulps exceeded 90% after 8 h of hydrolysis, outperforming “traditional” lignocellulosic biomasses such as miscanthus or sugarcane bagasse. Approximately 50 and 80 wt % of the protein were solubilized in [DMEtA][HCOO] and [DMBA][HSO_4_], respectively, while the remaining protein distributed between the pulp and lignin. However, the solubilized protein in the IL could not be recovered due to its low molecular weight. Regarding metal extraction, [DMEtA][HCOO] demonstrated higher efficiency, achieving 81% removal of Ni from *Lemna minor*’s pulps, whereas [DMBA][HSO_4_] extracted only 28% of Ni with slightly higher pulp concentrations. These findings indicate the need for further optimization in concurrent metal extraction using ILs.

## Abstract 2

This study explores an ionic liquid pretreatment for duckweed biomass to produce carbohydrates, such as cellulose and starch, and proteins and to enhance metal removal from contaminated feedstocks.

## 1 Introduction

The term “duckweed” refers to members of the *Lemnaceae* family, comprising around 40 species of small, floating macrophytes across 5 genera: *Lemna*, *Spirodela*, *Landoltia*, *Wolffia*, and *Wolfiella*.^1^ These species have attracted interest across biorefinery and wastewater remediation applications due to their highly distinctive combination of features: high growth rates,^2−5^ high starch and protein contents,^6−8^ and their ability to effectively remove high concentrations of nutrients (nitrogen and phosphorus^9,10^), BOD,^10−13^ and metals^14−16^ from wastewater.

Duckweed is primarily composed of cellulose (10–13%),^4,13,17−19^ starch (3–75%),^4,13,17^ proteins (7–45%),^4,13,17^ lipids (2–10%),^13,17^ lignin (2–9%),^4,13,17^ extractives (13%, only one value reported),^4^ and ash (12–28%).^4,13,17^ These fractions may be varied by manipulation of several environmental parameters. In general, duckweed species have been observed to accumulate starch during periods of environmental stress (e.g., nutrient deficiency, decreasing temperature, presence of heavy metals) as well as with increasing light intensity.^7,8,19−21^ Protein content and growth rate, however, have been observed to increase with increasing nutrient concentration and to decrease at low temperatures.^6,7^ Gupta et al. have suggested a trade-off between carbohydrate content and both protein content and growth rate, with the latter two increasing at the expense of the former when grown on high-nutrient water.^13^

There have been several studies looking at the performance of duckweed water remediation systems at laboratory-scale. These have mostly investigated the removal of nitrogen, phosphorus, and BOD from domestic wastewater using *Lemna minor*, *Spirodela polyrhiza*, and *Lemna gibba* (the most common species of duckweed). Typical removal rates within 10–20 days of retention are generally around 60–95% for nitrogen,^9,10,13,22−24^ 75–95% for phosphorus,^10,23−27^ and 70–95% for BOD,^10,25−27^ although these may reduce in colder climates which are thus less suited to such systems^10^ Of course, it is critical for any such process to consider any land use changes and other environmental effects, which can vary significantly with geography.^28^ There is very little data on the performance of full-scale duckweed remediation systems.^27,29^ In addition to nutrient and BOD removal, there have been a large number of studies investigating metal removal from wastewater, looking at the resulting concentration of metals within duckweed (and less commonly at the removal rate from the liquid solution).^15,16,30−36^ The advantage of phytoremediation for metal removal is due to the relatively high costs of conventional technologies (e.g. chemical precipitation, ion exchange, coagulation–flotation).^14,37^

Depending on the starch and protein content, duckweed can be utilized for different applications. If duckweed presents high starch content, low lignin content, small size, and ability to thrive in wastewater, it is a highly suitable candidate for bioethanol production.^5,38^ If, however, it presents a high protein content, it can be used as animal feed or as fertilizer due to its high water and nitrogen contents.^25,27^ Several studies have, nevertheless, been carried out on its use as a feedstock for production of bioethanol, with the volume of work on this topic increasing in the past decade.^4,5,18,21,39−44^ The generally high digestibility of duckweed relative to other bioethanol crops allows lower enzyme costs, which are said to lead to a lower production cost for ethanol than wheat straw.^45^ However, a pretreatment is required to further increase the enzymatic digestibility and thus reduce enzyme costs and energy inputs for grinding^2^

Protein extraction from biomass is of interest to improve the economic viability of biorefinery, to reduce the carbon footprint of nutritional protein, and for use in non-food applications such as coatings and polymers.^46−48^ Duckweed protein has been reported to have a favorable amino acid composition for supplementing human diets or to replace costly fish meal in aquaculture.^49−52^ Despite this, however, there has been only one process specifically developed for duckweed protein extraction (a 2015 patent), involving crushing and liquefaction of the feedstock, before addition of a coagulating agent, allowing recovery of a high-purity protein stream.^53^

Ionic liquids (ILs) have been used extensively for pretreatment of lignocellulosic biomass such as miscanthus, pine, birch chips. and wheat straw.^54−59^ These have targeted the fractionation of the biomass into a carbohydrate-rich stream (termed the pulp, composed of cellulose and sometimes hemicellulose) and a high purity lignin stream. Only two investigations (one paper, one patent) have been published involving the use of ionic liquids for duckweed processing; both aiming for the hydrolysis of the glucan through catalysis by an acidic ionic liquid (IL) (for production of platform chemicals or just direct hydrolysis to sugars).^60,61^ ILs have not been used to fractionate starch-rich biomass or to isolate protein from other biomass but have been studied for the processing of both pure starch as well as for the purification of proteins already extracted from biomass.

Biorefineries are facilities that can co-generate different bio-based products such as food, feed, materials, and chemicals and also bioenergy in a sustainable manner from biomass feedstocks. Lignocellulosic biorefineries are facilities that can co-generate different biorefineries focusing on the fractionation of lignocellulosic biomass by improving energy and water usage and combining biotechnological and chemical conversion routes.^62,63^

Global primary energy supply, as well as feedstocks in the chemical industry, are currently dominated by cheap and abundant fossil fuels.^64−66^ There is an increasing awareness of the serious environmental impact of both the CO_2_ emissions and the extraction of these fuels, which may manifest itself through carbon taxes that raise their price. However, cost-effective alternatives to fossil fuels are also required for a societal shift. The main alternatives that are put forward (such as wind, solar, or nuclear) are generally focused on carbon-neutral electricity generation. However, these technologies cannot directly solve the need for sustainable production of liquid fuels or platform chemicals, which require hydrocarbon inputs. These needs can only be feasibly met through biorefinery, the sustainable conversion of biomass to fuels, and high-value chemicals.^67^

In this study, the use of protic ILs for duckweed pretreatment is reported, aiming to utilize the feedstock for starch and protein accumulation and metal uptake and recovery. The overall aim was to therefore develop a single pretreatment process producing a highly digestible, starch-rich pulp, as well as a high-purity protein stream, with any heavy metals remaining dissolved in the IL. No previous studies have reported on IL pretreatment of duckweed, on IL pretreatments targeting protein extraction, or on combining bioethanol production with protein production and metal extraction in a single pretreatment process. Using time course pretreatments, the performance of two ILs was investigated for the pretreatment of two duckweed species in terms of starch recovery, sugar release, and protein recovery. In addition, the metal extraction capabilities of the ILs were evaluated for one batch of duckweed that was contaminated with heavy metals.

## 2 Materials and Methods

### 2.1 Feedstock and Chemicals

*Spirodela polyrhiza* was harvested from Regent’s Canal, London (Figure S1). *Lemna minor* was harvested from Holland Park, London. Following collection, all samples were rinsed with DI water, and any large fragments of non-duckweed material were removed by hand. *S. polyrhiza* samples were then spread out on paper towels and allowed to air dry over 1 week, before being stored away from sunlight in an airtight bag. After the samples were rinsed with DI water, a small fraction of the *L. minor* samples was air-dried on paper towels before being stored (as detailed above).

5 M sulfuric acid and 1-methylimidazole (>99.0%) were purchased from VWR; *N,N*-dimethylbutylamine (>98.0%) was purchased from Tokyo Chemicals Industry. Ethanolamine (>99%), acetic acid (>99%), *N,N*-dimethylethanolamine (>99.5%), formic acid (>98.0%), glucose (>99.5%), xylose (>99%), arabinose (>99%), α-amylase (>250 U/g), amyloglucosidase (>260 U/mL), potato, rice, corn, and wheat starch were purchased from Sigma-Aldrich, and 1 mol ampules of HCl were purchased from Fisher Scientific. Ctec2 enzymes were purchased from Novozymes (Bagsværd, Denmark). All the above were used as received for IL synthesis. Hoagland solution, CdCl_2_, and NiCl_2_ were all purchased from Sigma-Aldrich.

### 2.2 IL Synthesis and Characterization

Using a dropping funnel, acid was added dropwise to a round bottomed flask containing the corresponding amine, magnetically stirred, and chilled to 0 °C using an ice bath. IL water content was reduced to below 20 wt % by rotary evaporation and measured using a V20 Volumetric Karl Fischer Titrator (Mettler-Toledo). Acid–base ratios of [HSO_4_]^−^ ILs were determined by titration by 0.1 M NaOH, using a G20S Compact Titrator (Mettler-Toledo), standardized using potassium hydrogen phthalate purchased from Sigma-Aldrich. Acid–base ratios of [HCOO]^−^, [Cl]^−^, and [OAc]^−^ ILs were determined through calibration curves of acid–base ratio vs pH for dilute solutions (0.5 wt %). ^1^H-NMR and ^13^C-NMR spectra were recorded on a Bruker 400 MHz spectrometer (Supporting Information).

### 2.3 Pretreatment of Pure Starch

It was decided to first pretreat pure starch with a range of ILs. This was done for two reasons: first, to gain a better understanding of the future interactions between ILs and starch-based biomass; second, to identify conditions under which starch significantly degrades in various ILs and thus determine suitable initial duckweed pretreatment conditions. Four different types of starches were pretreated: potato, rice, corn, and wheat. Pretreatments were carried out at 90 and 120 °C for 30 min, with a water content of 20 wt % and at a solids loading of 10 wt %. A higher water content would have lower IL regeneration costs and IL viscosity but may also promote starch hydrolysis. The water content of 20 wt % was selected as a compromise between these factors and to allow easier comparison to results with lignocellulosic biomass.

**Figure 1:**
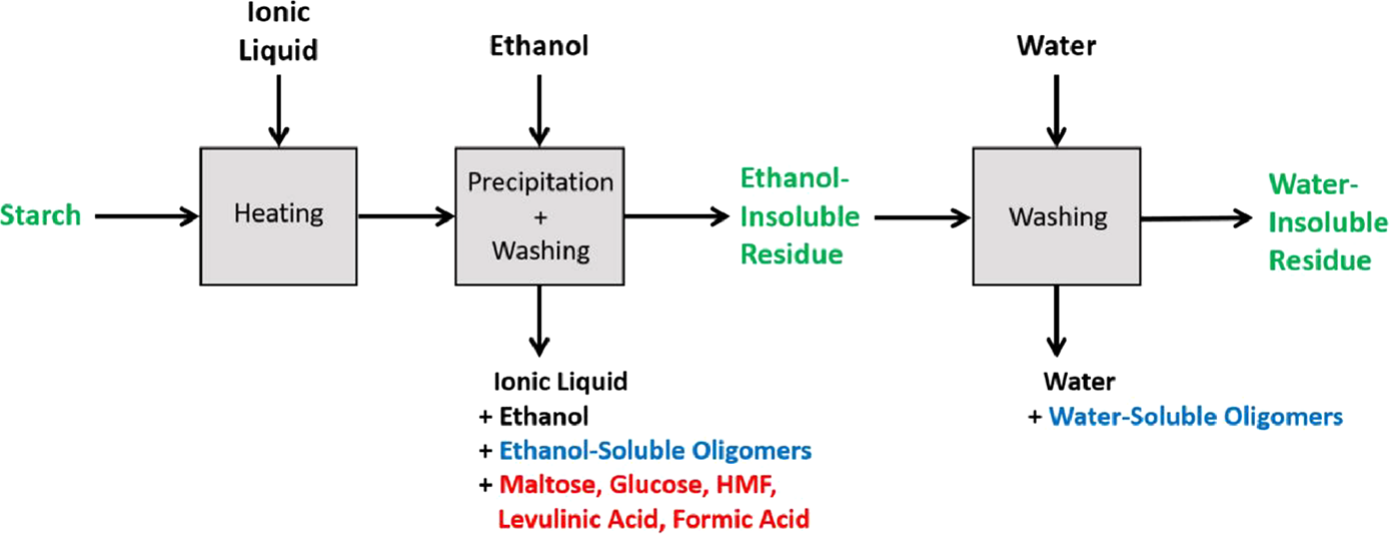
Material flow diagram during IL pretreatment of starch. Materials written in green are measured by mass, materials written in red are measurable by HPLC, and materials written in blue are not directly measurable.

Four protic ILs were selected for screening: *N,N*-dimethylbutylammonium hydrogen sulfate ([DMBA][HSO_4_]), 1-methyl imidazolium chloride [Hmim][Cl], dimethylethanolammonium formate ([DMEtA][HCOO]), and ethanolammonium acetate[MEA][OAc]. The first two of these are acidic ILs, whereas the two latter are neutral/basic ILs (as inferred from their pretreatment behaviors and aqueous pH). A mass balance was conducted for each condition tested, consisting of the recovered solid that was ethanol- and water-insoluble (termed the water-insoluble residue), the recovered solid that was ethanol-insoluble and water-soluble (termed the ethanol-insoluble residue), and the degraded solubilized material detected by HPLC (Figure 1). The purpose of isolating both the water-insoluble and ethanol-insoluble residues was to gain insight into the degree of partial depolymerization of the starch. Larger starch oligomers were expected to remain insoluble in water and ethanol, whereas smaller oligomers would be insoluble in ethanol but soluble in water.

### 2.4 Pretreatment of Biomass

**Figure 2:**
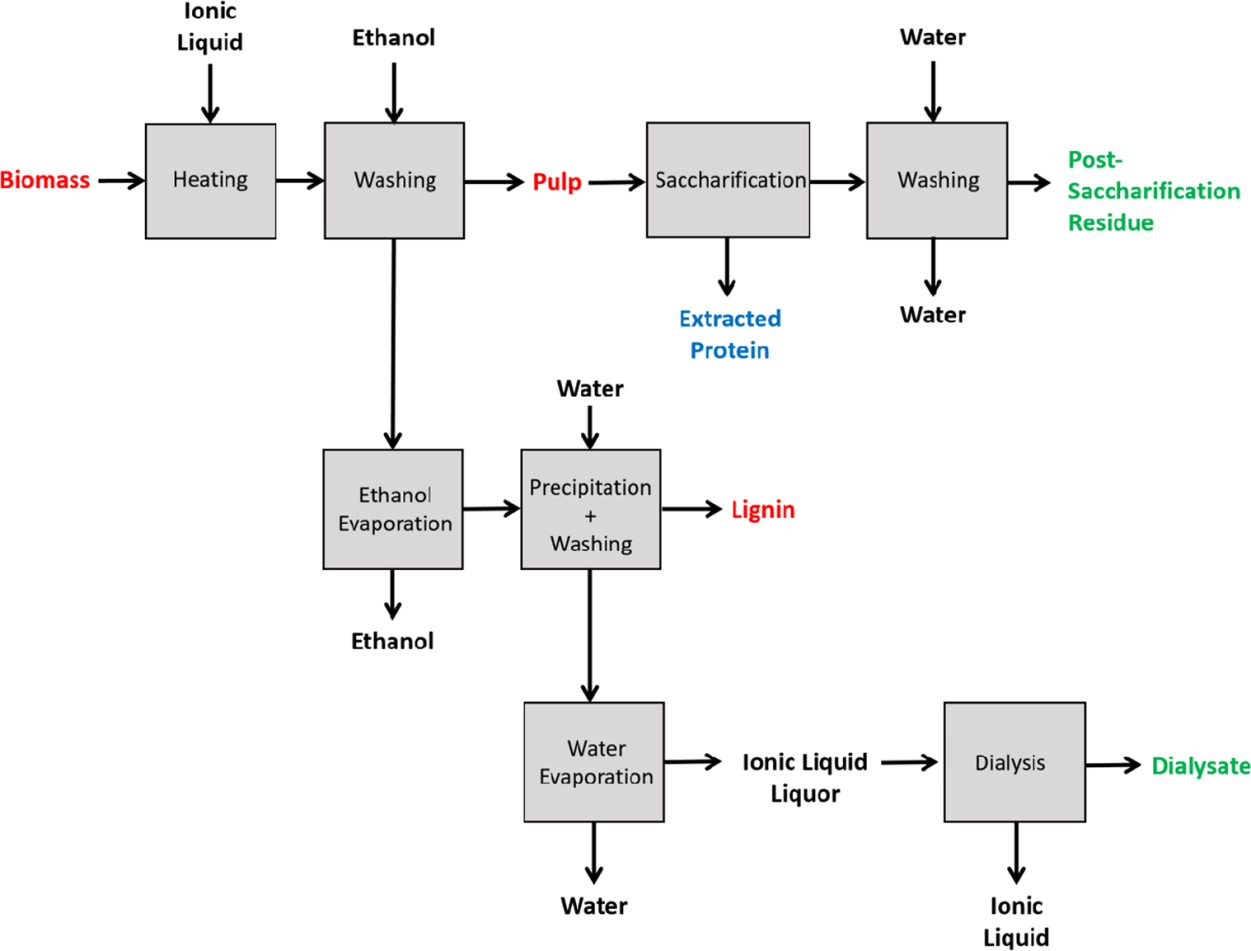
Protein fractionation during pretreatment. The protein content of the material written in red was measured through CHN (elemental) analysis for all samples. Protein content of the material written in green was measured through CHN analysis for selected samples. Protein content of the material written in blue was not directly measurable.

A workflow of the pretreatment process and its downstream is shown in Figure 2. *Spirodela polyrhiza* was pretreated according to the standard protocol of Gschwend (2017). Briefly, 1 g of biomass (in dry basis) was mixed with 9 g of the IL (20 wt % solids loading and 5:1 IL to water ratio) in a pressure tube at a certain temperature and time.^68^ Then, ethanol was added to the reaction slurry, and it was centrifuged. The supernatant containing IL, ethanol, and protein was decanted. The remaining pulp was air-dried and subjected later to enzymatic hydrolysis. The ethanol was evaporated from the liquid fraction by rotary evaporation, and water was added as the antisolvent to precipitate the solubilized lignin. No grinding of the biomass was carried out before pretreatment. Under milder pretreatment conditions, a portion of the pretreated duckweed floated following centrifugation during the first ethanol washing step. In such cases, the pulp was separated from the ethanol/IL mixture by vacuum filtration through a filter paper. The pretreatments consisted of time course experiments with [DMEtA][HCOO] at 120 °C and [DMBA][HSO_4_] at 90, 120, and 150 °C, with all timepoints carried out in triplicate. The aim of these pretreatments was to observe the effect on the duckweed composition and digestibility as well as on the partitioning of the protein fraction. An optimal pretreatment would both preserve starch in a highly digestible pulp and produce a high-purity protein fraction. However, it was anticipated that achieving both goals simultaneously may not prove feasible. Therefore, while the more prolonged pretreatments at 120 and 150 °C were expected to hydrolyze a substantial proportion of the starch, they were included in order to observe the effect of temperature on protein partitioning behavior. Full pretreatment and compositional analysis data for the pretreated samples are provided in the Supporting Information.

### 2.5 Enzymatic Hydrolysis

Enzymatic hydrolysis assays were carried out on pretreated pulps and untreated biomass as well as on blank controls. The main goal of the enzymatic saccharification was to evaluate the digestibility of the pretreated materials. A slightly modified version of the enzymatic hydrolysis protocol of the Hallett group laboratory was followed.^68^ Instead of 7 days, assays were carried out over 5 days. In addition to cellulase Ctec2 enzymes (Novozymes, Bagsværd, Denmark), α-amylase (>250 U/g) and amyloglucosidase (>260 U/mL) enzymes were added in order to hydrolyze the starch fraction.

Enzymatic hydrolysis assays were sampled after 8, 24, 48, 72, and 120 h of incubation. The hydrolysates were transferred into a microcentrifuge tube and centrifuged at 13.3*g* for 10 min in a VWR MICRO STAR 17R centrifuge, precooled to 4 °C. The supernatant was then filtered and run through an HPLC system (Shimadzu, Kyoto, Japan) with an Aminex HPX-87P column from Bio-rad (Hercules, USA), with purified water as a mobile phase at 0.6 mL/min and operating at a column temperature of 85 °C. All samples used were filtered through a 0.2 μm PTFE syringe filter. Calibration was carried out for each set of runs, using standards of 0.1, 1, 2, and 4 mg/mL glucose, xylose, mannose, galactose, and arabinose as well as 8 mg/mL glucose. A least squares linear regression fit was used to create a calibration curve. For selected samples, the solid residue from the saccharification assay was collected for protein content analysis.

### 2.6 Biomass Characterization

#### 2.6.1 Ash, Moisture Content, and Compositional Analysis of Lignocellulosic Materials

Determination of ash and moisture content and the compositional analysis protocol followed were that of the National Renewable Energy Laboratory.^69^ Unless specified, only ethanol Soxhlet extraction was performed on the pulp beforehand (as part of the pretreatment protocol), while water, ethanol, and hexane Soxhlet extractions were performed on the untreated biomass. All measurements were performed in triplicate on untreated biomass and once for each pulp. Cellulose content of the biomass was found by subtracting the starch content from the glucan content of the biomass.

#### 2.6.2 Determination of Protein Content

Biomass protein content was determined using a nitrogen-to-protein conversion factor of 6.25, a commonly used method that has also been shown to be accurate for duckweed.^70^ Biomass nitrogen content was measured by Medac Ltd. (Chobham, UK).

#### 2.6.3 Determination of Starch Content

Starch assays were carried out on pure starch, pretreated pulps (following separation and washing with absolute ethanol, as detailed in Section 2.4), and untreated biomass, as well as on blank controls. Starch content was determined using a modified version of the Megazyme Rapid Total Starch Assay procedure (using Note 1)^71^ in which released glucose was determined by HPLC rather than spectrophotometry. The HPLC (Shimadzu, Kyoto, Japan) was used with an Aminex HPX-87P column from Bio-rad (Hercules, USA), with purified water as a mobile phase at 0.6 mL/min and operating at a column temperature of 85 °C. All samples used were filtered through a 0.2 μm PTFE syringe filter. Calibration was carried out for each set of runs, using standards of 0.1, 1, 2, 4, and 8 mg/mL glucose. A least squares linear regression fit was used to create a calibration curve.

### 2.7 Biomass Digestion and Metal Quantification

Microwave-assisted acid digestion was conducted following EPA method 3050B with slight adjustments (Abouelela et al.^72^). Samples, including waste wood feedstock and IL liquor, were digested using nitric and hydrochloric acids in Teflon vessels. After microwave treatment, samples were diluted, filtered, and analyzed via ICP–MS (Agilent 7900, California, USA). Sludge-certified material was included for method validation. Analysis was conducted in triplicate, ensuring consistency and repeatability.

### 2.8 Liquor Dialysis

Dialysis was carried out on selected liquors in order to remove the IL and recover any dissolved protein. Dialysis tubing membranes with a 3.5 kDa molecular weight cut-off were purchased from Fisher Scientific and soaked in purified water for 5 min before use.

### 2.9 Metal Contamination of Duckweed

*L. minor* was collected from the wild and then transferred to water containing 2 ppm of Cd and Ni for 3 days. A small portion of the original duckweed was air-dried before being transferred into the metal-contaminated water in order to check the “baseline” level of these metals in the biomass. Following 3 days of growth in the contaminated water, the duckweed was harvested, washed with DI water, and air-dried. The washing with DI water was to ensure that any metals detected in the biomass were chemically bound to the biomass (either taken up or adsorbed to the surface) rather than remaining following evaporation of the metal-contaminated nutrient solution. A small portion of the metal-contaminated duckweed was air-dried without washing with DI water for comparison of metal contents. The complete procedure is shown in the Supporting Information.

### 2.10 Statistical Analysis

Statistical analysis was performed using the Student’s *t*-test for comparison of 2 data points and using two-tailed ANOVA followed by a posthoc Tukey test for comparison of three or more data points. Correlations were determined using Pearson’s correlation coefficient. When comparing saccharification curves, statistical analysis was applied to each sampling timepoint separately. The significance level used was 0.05, corresponding to a 5% risk of falsely concluding a statistical difference exists.

## 3 Calculations

Pulp yield was determined using eq 1:
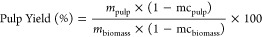
1where *m*_*x*_ is the mass of substance *x* added or recovered and mc is the moisture content of a solid on an air-dry basis. The subscript biomass refers to the untreated biomass used for pretreatment, and subscript pulp refers to the pretreated biomass.

Metal recovery in the pulp, lignin, and IL fractions were determined using eqs 2–4:
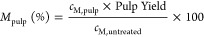
2
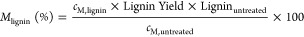
3
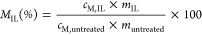
4where *c* is the concentration of metal M in the sample (determined by ICP-MS), Lignin is the proportion of lignin in the sample (the sum of acid-soluble and acid-insoluble lignin), and Pulp Yield is defined as in eq 1. Lignin Yield is the yield of recovered lignin (relative to the theoretical maximum), and *m* is the mass of a specified fraction. Subscript pulp refers to the pretreated biomass, untreated refers to the untreated biomass, IL refers to the recovered ionic liquid, and lignin refers to the recovered lignin. Metal removal from the pulp was then calculated as 100 – *M*_pulp_ (%), where *c*_sample_ is the glucose concentration of the hydrolysis sample as determined by HPLC, *c*_blank_ is the glucose concentration of the blank control as determined by HPLC, *V*_sample_ is the volume of the hydrolysis sample (10 mL), corr_anhydrous_ is a correction factor accounting for the increase in mass of sugars during hydrolysis (0.9 for glucose), Pulp Yield is as defined in eq 5, *m*_sample_ is the mass of sample hydrolyzed, mc_sample_ is the moisture content of the sample hydrolyzed, Glucan_untreated_ is the glucan content of the untreated biomass, and Glucan_pulp_ is the glucan content of the pulp.

### 3.1 Sugar Analysis

Equations 5–7 were used to calculate glucan content, starch recovery, and saccharification yield:
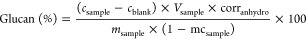
5
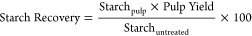
6
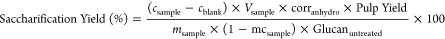
7where *c*_sample_ is the glucose concentration of the hydrolysis sample as determined by HPLC, *c*_blank_ is the glucose concentration of the blank control as determined by HPLC, *V*_sample_ is the volume of the hydrolysis sample (10.2 mL), corr_anhydrous_ is a correction factor accounting for the increase in mass of sugars during hydrolysis (0.9 for glucose), Pulp Yield is as defined in eq 1, *m*_sample_ is the mass of sample hydrolyzed, mc_sample_ is the moisture content of the sample hydrolyzed, Starch_untreated_ is the starch content of the untreated biomass, Glucan_untreated_ is the total glucan content of the untreated biomass (i.e., starch + cellulose), Starch_pulp_ is the starch content of the pulp, and Glucan_untreated_ is the total glucan content of the pulp. It should be noted that, although blank samples were analyzed in order to account for any glucose or starch contained in the enzyme and buffer solutions, they were found to contain no glucose during HPLC analysis. Equation 5 was used to calculate either starch content or total glucan content, depending on whether the sample was obtained using the *Determination of Starch Content* or *Compositional Analysis* protocols.

## 4 Results and Discussion

### 4.1 Pretreatment of Virgin Duckweed

#### 4.1.1 Duckweed Composition

The composition of the untreated duckweed was cellulose, 7.3 wt % ± 0.3 wt %; starch, 3.2 wt % ± 0.1 wt %; hemicellulose, 6.3 wt % ± 0.1 wt %; lignin, 13.9 wt % ± 0.3 wt %; protein, 35.0 wt % ± 0.7 wt %; ash, 16.2 wt % ± 0.9 wt %; extractives, 20.5 wt % ± 2.9 wt %. According to the literature, duckweed tends to rapidly accumulate starch during periods of stress, rather than periods of rapid growth in favorable conditions.^6−8,73^ The duckweed was harvested during a period of high temperatures and consistent sunlight and was thus growing extensively, covering the water surface. The glucan content (and particularly starch content) of the feedstock is thus fairly low, while the protein content is high (higher than the large majority of agro-residues considered for protein extraction^46^ but at the top of the reported range for duckweed).^52,74,75^

#### 4.1.2 Starch Solubilization

**Figure 3:**
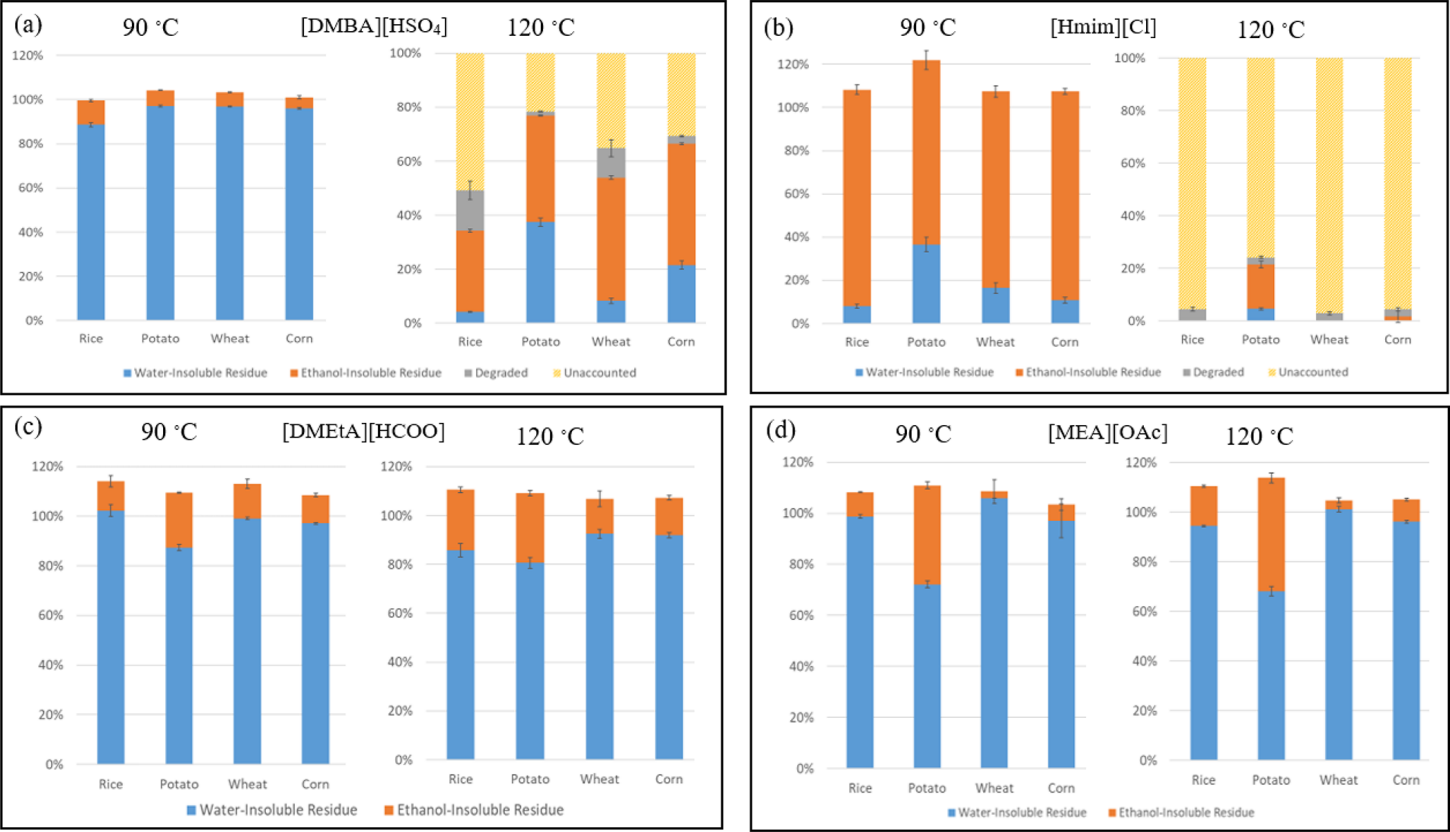
Fractionation of different starches following pretreatment with (a) [DMBA][HSO_4_], (b) [Hmim][Cl], (c) [DMEtA][HCOO], and (d) [MEA][OAc]. All pretreatments were carried out at a solids loading of 10 wt %, with an IL water content of 20 wt %, for a duration of 30 min.

The study initially focused on pretreating pure starch with various ionic liquids (ILs) to understand their interactions with starch-based biomass and to determine suitable pretreatment conditions. Four types of starch (potato, rice, corn, and wheat) were pretreated at 90 and 120 °C with different ILs, considering factors like water content and solid loading. Protic ILs ([DMBA][HSO_4_], [Hmim][Cl], [DMEtA][HCOO], and [MEA][OAc]) were selected, and a mass balance analysis was conducted to assess starch degradation and residue formation (Figure 3). The choice of starch types aimed to investigate the effects of IL pretreatment across varied starch properties, such as granule size and molecular weight. Although hydrolysis of starch was one of the ultimate aims of the pretreatment, hydrolysis within the IL presents both recovery challenges and risks of degradation of the glucose. Results indicated varying degrees of starch dissolution and degradation across different ILs and temperatures. For instance, [DMBA][HSO_4_] showed limited starch dissolution and minimal degradation at 90 °C but significant degradation at 120 °C. On the other hand, carboxylate ILs ([DMEtA][HCOO] and [MEA][OAc]) had similar results and fully dissolved starch without extensive depolymerization, indicating potential as effective solvents for starch pretreatment. However, mass balance discrepancies suggested residual ILs and possible starch retrogradation during recovery. Similarly, [Hmim][Cl] effectively dissolved starch, leading to substantial depolymerization and low residue yields, especially at higher temperatures, making it unsuitable for starch pretreatment due to its acidity. [DMBA][HSO_4_] and [DMEtA][HCOO] were chosen for the pretreatment assays due to their different acidities (carboxylate versus hydrogen sulfate) and, depending on the conditions, starch had limited degradation.

#### 4.1.3 Starch Recovery

**Figure 4:**
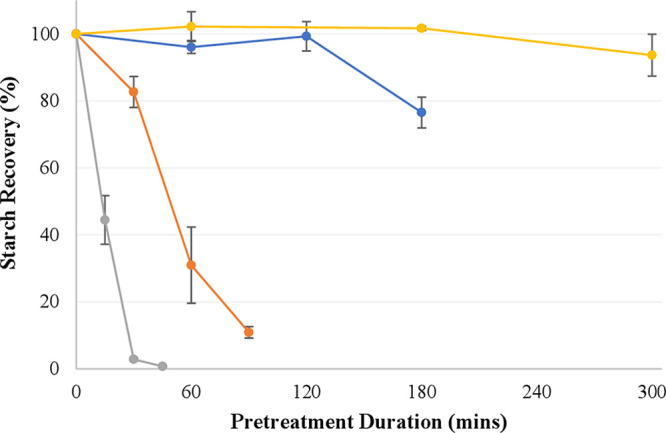
Starch recovery in *Spirodela polyrhiza* under several pretreatment conditions. In yellow, [DMEtA][HCOO] at 120 °C; in blue, [DMBA][HSO_4_] at 90 °C; in orange, [DMBA][HSO_4_] at 120 °C; and in gray, [DMBA][HSO_4_] at 150 °C. All pretreatments were carried out with 20 wt % water and at a biomass loading of 10 wt %.

Full pretreatment and compositional analysis datasets are provided in the Supporting Information. Starch recoveries (i.e., starch recovered from the pretreated pulp) under all four conditions are displayed in Figure 4. Based on the results using pure starch (Figure 3), it was expected that high starch recoveries would be observed using [DMEtA][HCOO], as well as [DMBA][HSO_4_] at 90 °C. Above 90 °C, the highly acidic [DMBA][HSO_4_] was expected to hydrolyze the starch rapidly. The results using duckweed align closely with this predicted behavior.

Using [DMEtA][HCOO], quantitative starch recovery is observed between 1 and 3 h, with the slight decrease at 5 h found not to be statistically significant. This is somewhat in line with observations using pure starch (Supporting Information), in which raising the temperature from 90 °C to 120 °C led to a slight decrease in water-insoluble residue (5–16 wt % depending on starch type), showing that depolymerization of the starch was occurring, albeit at a slow rate.

Using [DMBA][HSO_4_], results were found to be highly dependent on the temperature. At 90 °C, near-quantitative recoveries were observed up to 2 h. However, a significant drop occurred after 3 h, with recoveries decreasing to 77%. It would, therefore, appear that the starch is depolymerizing at 90 °C, but at a slow rate. Initially, the resulting starch fragments remain insoluble in the IL/ethanol mixture but begin to solubilize between 2 and 3 h. Expectedly, increasing the temperature accelerates the kinetics of this depolymerization, with no quantitative recoveries observed at any of the measured timepoints at either 120 or 150 °C. After 30 min at 150 °C, near-quantitative removal of the starch has occurred.

#### 4.1.4 Saccharification

**Figure 5:**
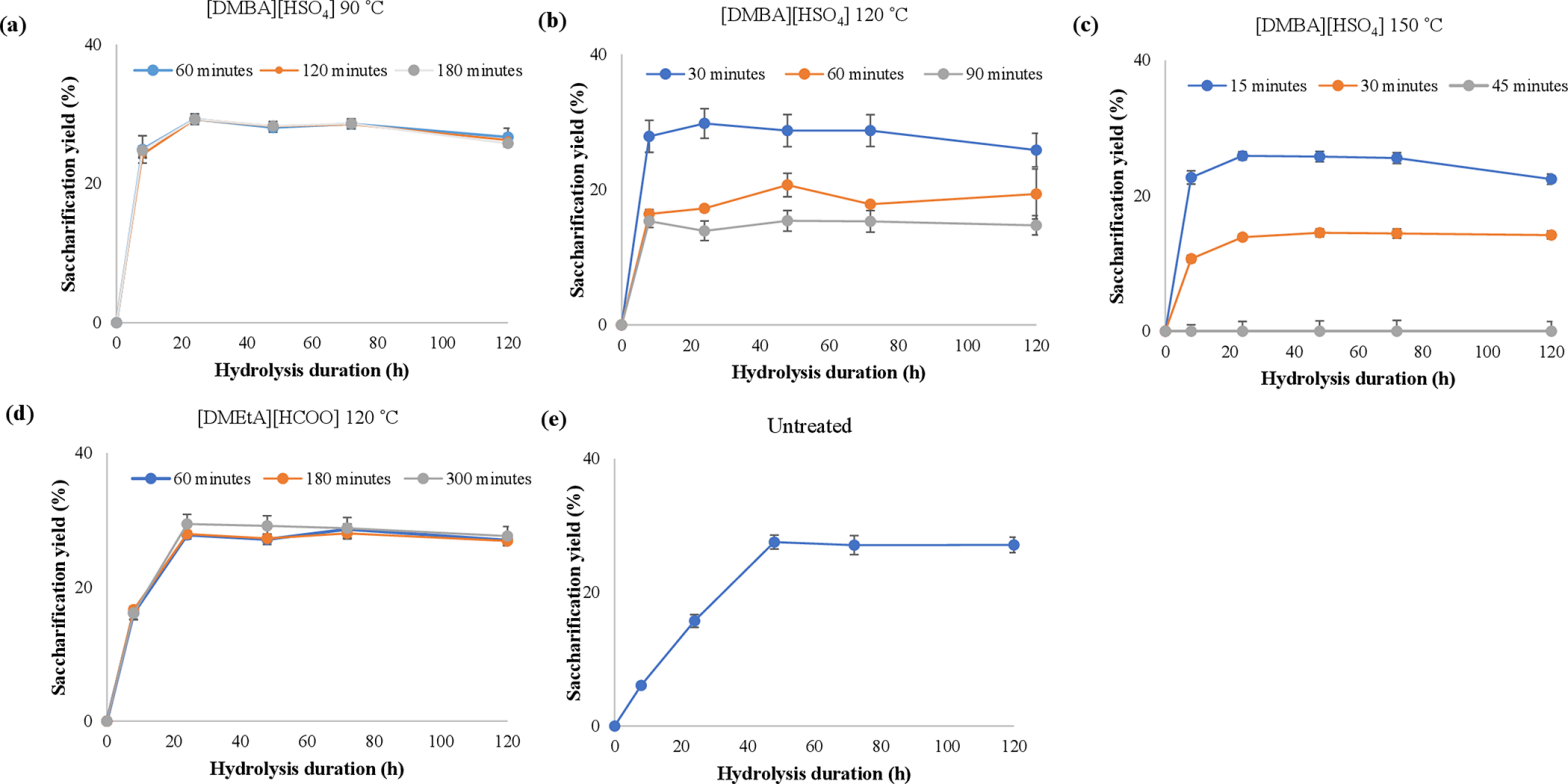
Xylose yields for the saccharification kinetics of pulps pretreated using varying ionic liquids and temperatures. (a) [DMBA][HSO_4_] at 90 °C; (b) [DMBA][HSO_4_] at 120 °C; (c) [DMBA][HSO_4_] at 150 °C; (d) [DMEtA][HCOO] at 120 °C. (e) Untreated biomass. All pretreatments were carried out on *Spirodela polyrhiza*, with 20 wt % water, and at a biomass loading of 10 wt %. It should be noted that saccharification yield and digestibility are numerically equivalent in untreated biomass.

**Figure 6:**
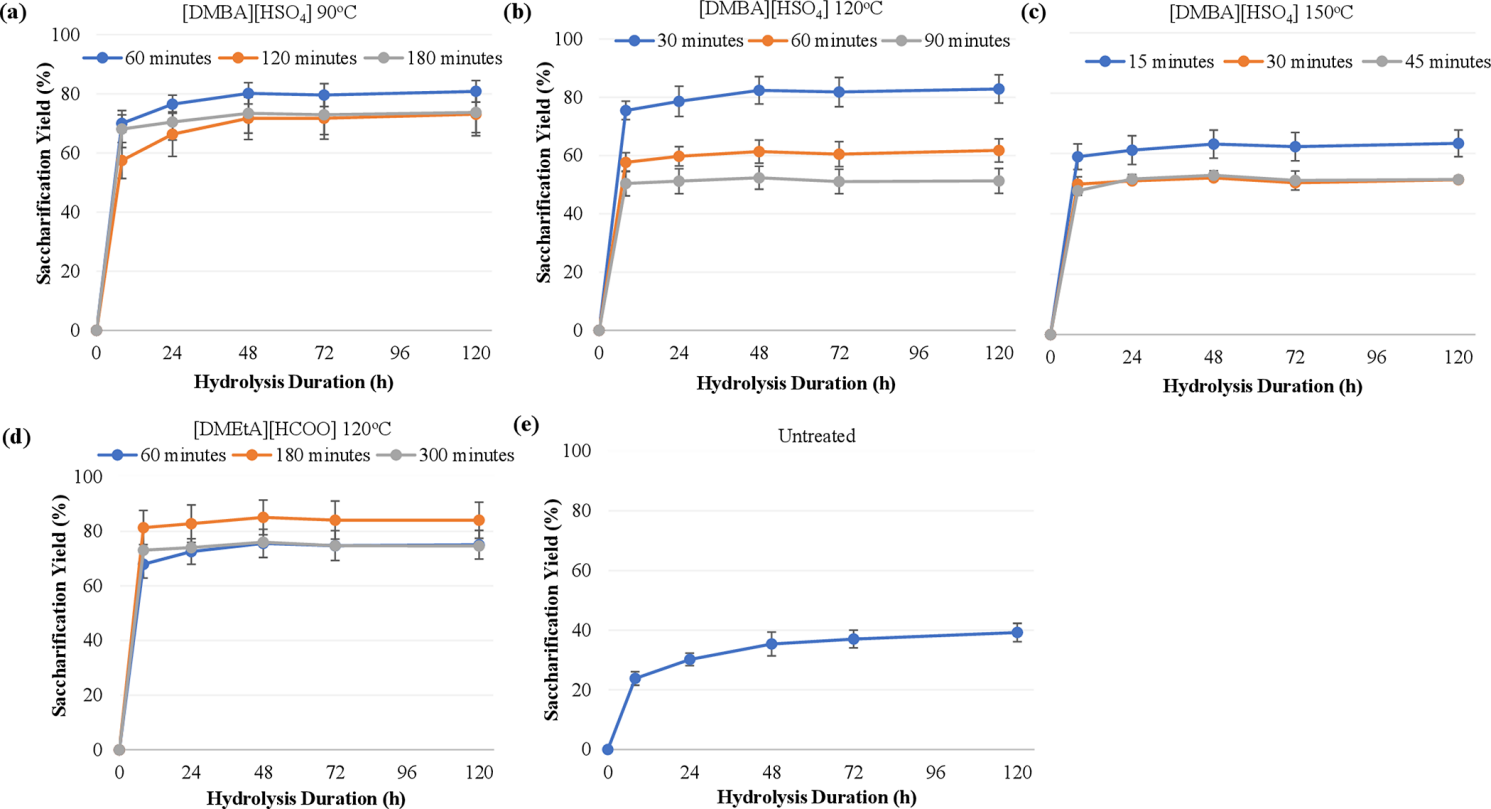
Glucose yields for the saccharification kinetics of pulps pretreated using varying ionic liquids and temperatures. (a) [DMBA][HSO_4_] at 90 °C; (b) [DMBA][HSO_4_] at 120 °C; (c) [DMBA][HSO_4_] at 150 °C; (d) [DMEtA][HCOO] at 120 °C; (e) Untreated biomass. All pretreatments were carried out on *Spirodela polyrhiza*, with 20 wt % water, and at a biomass loading of 10 wt %. It should be noted that the saccharification yield and digestibility are numerically equivalent in untreated biomass.

Results for the enzymatic saccharification of the untreated and pretreated materials in terms of xylose and glucose yields are presented in Figures 5 and 6. The relatively large variances displayed were presumed to be due to heterogeneity in the biomass (harvested from a canal). As a result, statistical analysis was used to determine whether differences between samples were statistically significant and to what degree.

##### 4.1.4.1 Xylose Yields

Mild pretreatment conditions for [DMBA][HSO_4_] such as all assays conducted at 90 °C (Figure 5a) and 120 °C for 30 min (Figure 5b) produced slightly higher xylose yields than those of the raw feedstock control (Figure 4e). Once [DMEtA][HCOO] pretreatment is inherently milder than that of [DMBA][HSO_4_], the xylose yields (Figure 5d) were also higher than those of the raw feedstock control (Figure 5e). Surprisingly, the yields did not exceed 30%, which indicates the remainder hemicellulose was more recalcitrant to enzyme accessibility.

##### 4.1.4.2 Glucose Yields

For all pulps (Figure 6a–d) except those pretreated for 1–2 h at 90 °C, saccharification (taking into account both starch and cellulose) was over 90% complete after 8 h of hydrolysis. This is substantially faster than observed using more “traditional” lignocellulosic biomass such as miscanthus or sugarcane bagasse, which have been show to take around 24–72 h to reach full near-maximum yield under the similar enzyme loadings.^68,76−78^ Such accelerated rates are extremely important, allowing for a potential trade-off between enzyme loading and hydrolysis duration. As pretreatment severity increased, hydrolysis kinetics tended to increase slightly, likely as a result of increased disruption of the biomass structure and, thus, higher substrate accessibility. Untreated biomass displayed significantly slower kinetics, taking 48 h to reach 90% of its final value.

Pretreatment using [DMEtA][HCOO], at all conditions (Figure 6d), significantly enhanced both saccharification yield relative to untreated biomass and hydrolysis kinetics. Particularly for 180 and 300 min of pretreatment, pulps displayed very rapid hydrolysis kinetics, with 97% and 98% of the final yield reached after 8 h of hydrolysis. Statistical analysis revealed that the difference between all three conditions was only significant after 8 h of hydrolysis. At longer hydrolysis durations, all three conditions are not statistically different, as the slower kinetics of the less severely pretreated samples eventually converge.

Saccharification behavior using [DMBA][HSO_4_] was, again, found to be dependent on pretreatment temperature. At 90 °C, pulp saccharification was found to be substantially better than untreated biomass at all three durations, despite relatively little apparent disruption to the biomass structure. At 120 °C, pulps pretreated using [DMBA][HSO_4_] display more variability in results. The shortest pretreatment duration of 30 min is the optimal condition for saccharification yield at all hydrolysis timepoints, with similar results to those using [DMEtA][HCOO] and [DMBA][HSO_4_] at 90 °C. At 150 °C, trends in saccharification yields are similar to those at 120 °C, despite being substantially lower (51–63% after 120 h of hydrolysis). The reduction in saccharification yields over time using [DMBA][HSO_4_] at elevated temperatures is due to glucan degradation, limiting saccharification yields.

The optimal operating conditions for sugar release were thus determined to be [DMEtA][HCOO] for 180 min and using [DMBA][HSO_4_] for 30 min at 120 °C. At these conditions, saccharification yields of 76–81% were achieved after 8 h of hydrolysis, rising to 83–84% after 120 h. This highlights the potential of duckweed for bioethanol production: these yields are similar to, or higher than, traditional lignocellulosic energy crops (such as *Miscanthus*, willow, sugarcane bagasse, or waste wood) pretreated using protic ILs.^54,72,79,80^ This is despite much milder conditions and shorter hydrolysis durations. While it must be noted that the carbohydrate content of the *S. polyrhiza* strain used is much lower than that of most feedstocks investigated for bioenergy, enzyme loadings were adjusted correspondingly, and starch recovery results were in line with those obtained using pure starch (see Supporting Information). Similar trends and results can thus be expected with strains containing higher carbohydrate contents.

Comparison with other duckweed pretreatment studies is challenging for a number of reasons. Many studies focus on bioethanol production using either simultaneous saccharification and fermentation configurations or sequential. In such cases, the hydrolysis performance is often not evaluated in great detail, instead focussing on ethanol yield.^4,41,43,81^ The second main reason comparison is challenging is the lack of reporting of enzyme loadings, the use of differing loadings, or the use of different classes of enzymes (e.g., pullulanases). This is also not helped by a lack of “control” hydrolysis on completely untreated duckweed which would allow the relative improvements in digestibility to be quantified, missing from almost all investigations except those of Zhao et al.^18,82^ Despite this, results obtained here appear in line with those of other such studies.^4,5,39,40,42,83^

#### 4.1.5 Protein Partitioning

**Figure 7:**
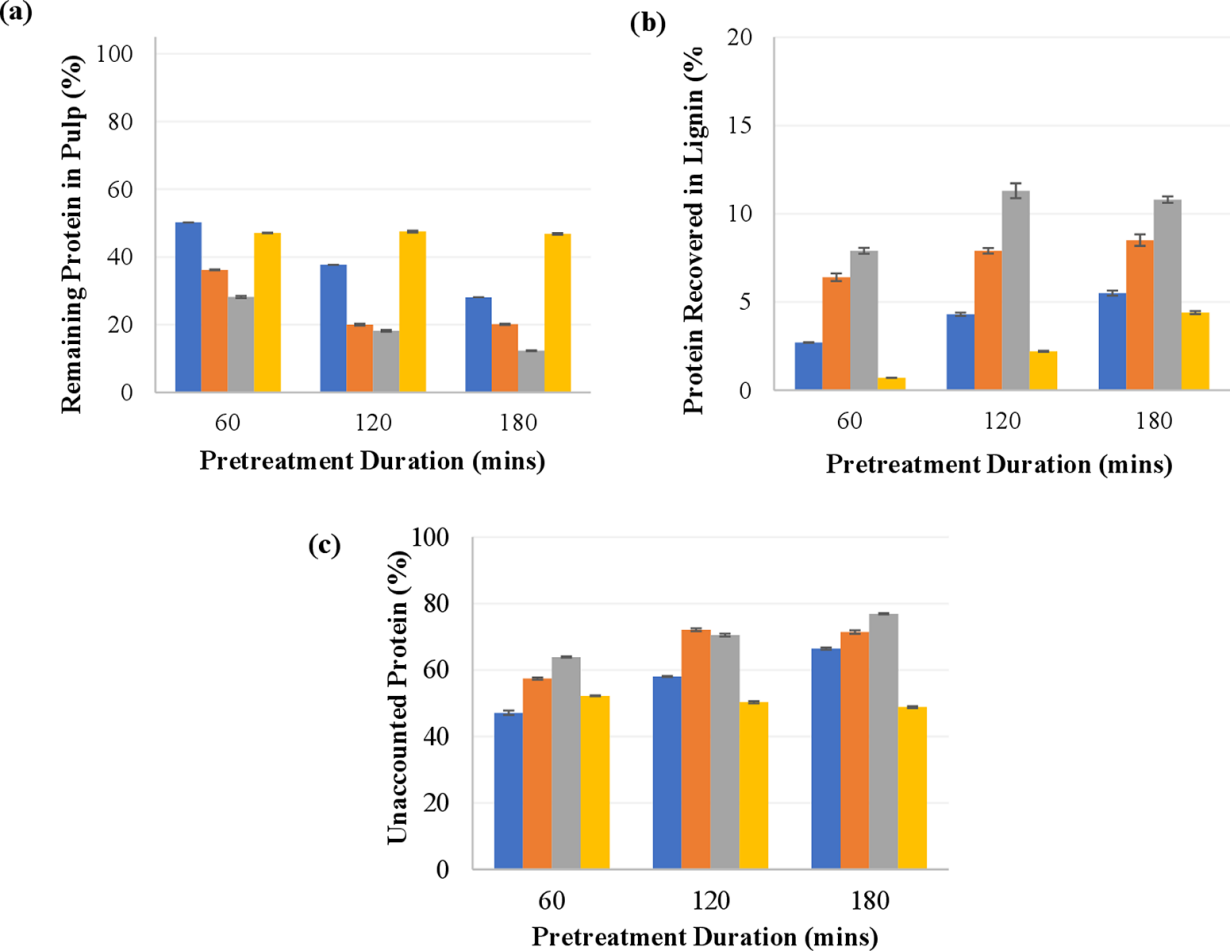
Protein partitioning during IL pretreatment of *Spirodela polyrhiza*. (a) Remaining protein in the pulp after pre-treatment. (b) Protein content in lignin. (c) Unaccounted protein refers to remaining protein not detected in the pulp or lignin fractions. In yellow, [DMEtA][HCOO] at 120 °C; in blue, [DMBA][HSO_4_] at 90 °C; in orange, [DMBA][HSO_4_] at 120 °C; and in gray, [DMBA][HSO_4_] at 150 °C. All pretreatments were carried out with 20 wt % water and at a biomass loading of 10 wt%.

The material flow diagram depicting the protein fractionation during pretreatment is in Figure 1. The protein content of the pulp and lignin streams was determined for all conditions, and a mass balance was thus carried out, with results displayed in Figure 7. Neither the lignin nor the pulp fractions were found to have enriched protein contents relative to those of the untreated biomass (see the Supporting Information for numerical data). Using [DMBA][HSO_4_], the protein content of the pulp decreased with pretreatment duration, while the protein content of the lignin fraction increased. Using [DMEtA][HCOO], protein content stayed constant at around 25% in both the pulp and the lignin fractions.

The behavior of both ILs is fairly distinct regarding protein removal from the pulp. Using [DMBA][HSO_4_], protein was selectively removed from the pulp. Trends were very similar across temperatures, with kinetics accelerated at higher temperatures Although the rate of removal was observed to decrease over time at a given temperature, there was no clear sign of a plateau, as with [DMEtA][HCOO]. This may be a sign of proteins being broken down and dissolved into the IL: the peptide bonds linking the amino acids that make up proteins can be hydrolyzed, with this reaction strongly catalyzed by acidic or basic conditions, as well as temperature.^84−86^ Protein yield in the lignin fraction tended to increase over time due to both increasing lignin yields and an increasing protein content. However, due to the low quantities of recovered lignin, this yield is fairly low (reaching a maximum of 11% of starting protein).

Using [DMEtA][HCOO], approximately half the protein was removed from the biomass by 60 min of pretreatment, and this value did not change substantially over time. This may be due to the relatively low degree of disruption of the biomass using this IL, as well as its less acidic nature, leading to a lower degree of protein hydrolysis. The extracted fraction may also correspond to the water-soluble fraction of leaf protein, which is typically between 40% and 50%.^87^ The amount of protein removed from the pulp using [DMEtA][HCOO] is also comparable to that removed by alkali extraction at pH 8.5 from *S. polyrhiza* in a study by Yu et al.^74^ The protein content in the lignin fraction increased consistently over time due to increasing lignin yields rather than due to enrichment. As lignin yields were very low during the pretreatment (due to the slower pretreatment kinetics of [DMEtA][HCOO] and the low lignin content in the biomass), protein yields in this fraction were thus below 5%.

Under all conditions, the total mass balance from the pulp and lignin fractions was very low, with 49–77% of the original protein being unaccounted. It was presumed that this fraction must be contained within the IL liquor. In order to recover any dissolved protein, dialysis was carried out on selected pretreatment liquors: 60 and 180 min using [DMEtA][HCOO] and 30 and 60 min using [DMBA][HSO_4_] at 120 °C. The resulting solution was then freeze-dried. The recovered solids were paler in appearance (Supporting Information) but were found to only have a moderately increased protein content at most conditions: 23% and 49% for 60 and 180 min using [DMEtA][HCOO] and 46% and 53% for 30 and 60 min using [DMBA][HSO_4_]. However, the recovered solid only accounted for 2–5% of the starting protein, and thus did not significantly affect the overall protein mass balance. One of the reasons related to the large unaccounted protein fraction is that proteins dissolved in the IL are being hydrolyzed into oligopeptides/amino acids that are too small to be recovered by dialysis. If such oligopeptides were recoverable (e.g., by electrophoresis or using a smaller membrane pore size), the mass and purity of the resulting solid fraction would thus be expected to increase substantially.

Pulps obtained following pretreatment with [DMEtA][HCOO] were found to contain a large fraction of the protein in the starting biomass, albeit at low purity, and demonstrated promising saccharification performance. It was therefore hoped that saccharification would lead to a larger concentration of protein in the solid fraction. Following saccharification, the solid residue was collected, washed with DI water (in order to remove any enzymes), and freeze-dried. This residue was found to have a moderately increased protein content relative to the pulp: 36%, 46%, and 41% in pulps pretreated for 60, 180, and 300 min. This was equivalent to a yield of 97%, 71%, and 70% relative to the protein contained within the corresponding pulps or 46%, 34%, and 33% of the original protein.

While these protein partitioning results may not seem that promising at first glance, comparison with previous studies shows a possible cause for optimism, albeit one that will require further study. Firstly, should the oligopeptides in the IL liquor be recoverable, a high purity protein fraction would be expected, with the reduced molecular weight being a desirable property for nutritional applications.^88^ This would be the ideal outcome due to the high purity of such a stream but depending on the degree of hydrolysis of the protein may not be feasible. It would be similar to the protein extraction process reported by Wahlström et al., who used urea and carboxylate-based Deep Eutectic Solvents (DESs) to extract protein from Brewer’s Spent Grain (BSG), a common high-protein waste product from the brewing process.^89^ 80% of proteins were extracted after 1 h at 80 °C and were recovered by dialysis to a purity of around 50% (containing both polysaccharides and lignin). A 37% loss was observed between extraction and solid recovery, attributed to protein fragmentation (to below the molecular weight cut-off of 3.5 kDa) as in this study.

Secondly, compared to a number of other studies attempting to integrate bioethanol production with protein extraction, the final protein concentrations in the post-saccharification residue are relatively elevated; the high starting protein concentrations in the duckweed also mean the absolute yields may still be reasonable, particularly for valorization as a secondary co-product. For example, Hou et al. increased protein concentration in brown seaweed from 4% to 14% (with a 97% yield) in post-saccharification residue following a simple milling pretreatment.^90^ Similarly, Hamley-Bennett et al. reported an increase in protein content from 8% to 15% following steam pretreatment and saccharification of sugar beet (although no yield was provided).^91^

#### 4.1.6 Fate of Hemicellulose

Two different types of protic ionic liquids have been probed, one alkaline and one acidic ([DMEtA][HCOO] and [DMBA][HSO_4_]), with different reactivities toward hemicellulose solubilization. The more acidic ionic liquid, [DMBA][HSO_4_], solubilizes most of the hemicellulose, and the pretreated materials present a high cellulose content. However, the hemicellulose oligomers and monomers cannot be easily recovered from the ionic liquid, which results in economic loss of this valuable fraction. On the other hand, the more alkaline ionic liquid, [DMEtA][HCOO], solubilizes less hemicelluloses and the pretreated materials present both cellulose and hemicellulose which can then be enzymatically hydrolyzed into a pentose and hexose liquor. This liquor can then be fermented by pentose/hexose fermenting microorganisms (either wild type yeasts such as *Kluveromyces Marxianus* or *Scheffersomyces stipitis* or engineered *Saccharomyces cerevisiae*) into second-generation bioethanol.^92^

### 4.2 Pretreatment of Metal-Contaminated Duckweed

Metal-contaminated *Lemna minor* was pretreated using [DMEtA][HCOO] at 120 °C for 180 min and [DMBA][HSO_4_] at 120 °C for 30 min. These conditions were previously identified as the most promising for sugar release. The aim of these experiments was 2-fold: first, to observe whether pretreatment behavior is similar between different batches of duckweed; and second, to determine whether these promising pretreatment conditions are suitable for metal removal. Changing both the species and the exact location of harvest was done to replicate the realistic variation in input feedstocks, which could enter a biorefinery. Duckweed species can be very difficult to visually distinguish without expert guidance; so, mixed-species input feedstocks would be expected, and composition can vary substantially by geography due to differing growth conditions. Fractionation performance was found to be very similar to that of *S. polyrhiza* despite the starting material having a higher glucan content, lower protein content, and being metal contaminated (see Supporting Information). This is encouraging evidence for these process conditions being relatively feedstock-independent in terms of duckweed strain, composition, and degree of metal contamination.

The composition of the untreated duckweed was as follows: cellulose, 10.4% ± 0.5 wt %; starch, 10.0 wt % ± 0.1 wt %; hemicellulose, 5.7 wt % ±0.1 wt %; lignin, 6.7 wt % ±0.1 wt %; protein, ash, 19.9 wt % ±0.5 wt %; extractives, 32.1 wt % ±0.7 wt %; and unaccounted, 9.7 wt % ± 0.5 wt %. Compared to *S. polyrhiza*, this strain of duckweed was found to have a substantially lower protein content and higher starch content. This is consistent with literature: duckweed has been reported to increase starch content and reduce protein content when in a stressed environment (such as heavy metal-contaminated water).^6,13,93,94^ The unaccounted 10 wt % of the mass is thus due to either other extractable materials that solubilize during compositional analysis (e.g., apiose or pectins)^39^ or the assumed nitrogen/protein conversion factor not being appropriate for this feedstock.

#### 4.2.1 Metal Uptake and Extraction

Results for the metal content of the biomass before and after growth in metal-contaminated water are displayed in Table 1. The original biomass was found to contain no Cd and only 9 ppm of Ni. Following growth in a metal-contaminated solution, high levels of both metals were observed in the biomass. The contents are generally lower than those reported in literature for duckweed grown in similar concentrations of Cd (around 2000–6000 ppm), possibly due to the shorter duration.^95,96^ For duckweed grown in Ni, mixed values have been reported in previous studies, ranging from no uptake at water concentrations of 0.23–2 ppm in water but tissue concentrations of 800–3000 ppm at 5 ppm water concentrations^15,97^ to 15 ppm tissue concentrations from 0.06 ppm in water.^21^ This may be taken as an example of the strong variability of individual duckweed strains. Rinsing with DI water was found to remove only a small proportion of the metals, showing that the metals were either taken up or chemically bound to the surface of the biomass.

**Table 1:** Metal Contents of Duckweed before and after Growth in Metal-Contaminated Water

feedstock	Ni (ppm)	Cd (ppm)
Virgin	9 ± 13	0 ± 0
Contaminated	453 ± 41	681 ± 62
Contaminated + DI water rinse	414 ± 29	636 ± 3

Although the effect on growth was not investigated, these results are further evidence of the ability of duckweed to uptake heavy metals in significant quantities and confirm that it has significant potential for phytoremediation of water. Some studies focusing on the physiological responses of duckweed to heavy metals over similar concentration ranges have shown negative impacts such as decreased photosynthetic pigments and decreased protein contents.^16,93,98−101^ It is worth noting, however, that for some metals conflicting findings have been reported on metal toxicity limits, likely due to differences in tolerance between strains, as well as increasing tolerance from higher nitrogen and phosphorus concentrations.^36^ Therefore, further work should be performed to establish clearer limits for the application of duckweed for metal removal. This should focus on whether duckweed can be used for high levels of metal contamination (i.e., several mg/L) or whether it should only be used for “polishing” applications with low metal concentrations. Further studies could involve the use of different configurations (e.g., a low-concentration “pool” for duckweed growth before transplantation to a high-concentration “pool” for rapid accumulation before harvesting) or genetic modification of duckweed for heavy metal tolerance (with genetic modification already of interest due to duckweed’s relative biological simplicity).^102^

**Figure 8:**
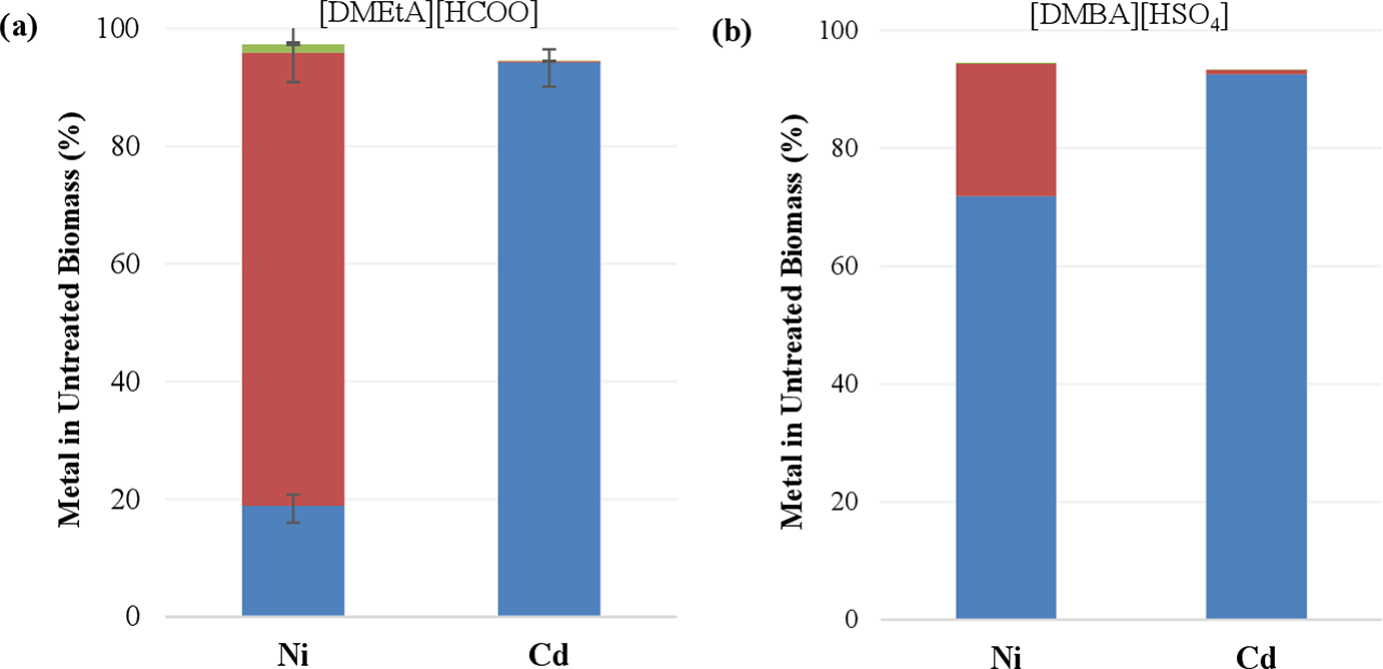
Metal partitioning during IL pretreatment of metal-contaminated *Lemna minor* in (a) [DMEtA][HCOO] and (b) [DMBA][HSO_4_]. In blue: pulp; in green: lignin; in red: liquor. All pretreatments were carried out at 120 °C, with 20 wt % water, and at a biomass loading of 10 wt %. Pretreatments were carried out for a duration of 30 min using [DMBA][HSO_4_] and 180 min using [DMEtA][HCOO].

The metal contents of the pulp, lignin, and liquor were then determined following pretreatment, allowing the partitioning behavior to be determined. Results are displayed in Figure 8. Large differences were observed in the partitioning behavior of both metals, as well as moderate differences between the performance of both ILs. Metals were predominantly concentrated in the pulp and liquor fractions, with the lignin fractions displaying both low absolute quantities and concentrations of either metal.

Ni extraction proved to be more successful out of the two metals, particularly using [DMEtA][HCOO]. This IL achieved 81% removal of Ni from the pulp. As a result, pulp concentrations dropped to under 100 ppm from over 400 ppm in the starting biomass. Using [DMBA][HSO_4_], only 28% of the Ni was extracted from the pulp, with resulting pulp concentrations slightly higher than those of the starting biomass. Cd extraction proved unsuccessful in both ILs, with only 6–7% removal and with pulps having significantly higher Cd concentrations than the starting biomass. In this case, the pretreatment process essentially concentrated the metal in the remaining solid fraction.

The reasons behind the generally unsuccessful metal extractions are not completely clear. [DMBA][HSO_4_] was shown to remove around 65% of the Cd in metal-contaminated waste wood in the work of Abouelela et al., in which a protic alkylammonium carboxylate IL showed similar extraction capabilities for Cd.^72^ However, the starting material contained only 1 ppm of the metal, and therefore, absolute quantities extracted were therefore small. In the same study by Abouelela et al., Ni extraction was also measured (with an original concentration of 8 ppm), with both ILs extracting around 50–60% of the Ni present.

The most likely hypothesis for the generally low levels of extraction is that these metal species have a limited affinity for the ILs employed (with the exception being Ni in [DMEtA][HCOO]) and that, in order for them to be extracted, the biomass structure would need to be severely disrupted by employing harsher pretreatment conditions. [Hmim][Cl] was not considered for duckweed pretreatment in this study due to its poor performance when pure starch was pretreated (data not shown). However, it has been shown to outperform a number of acidic ILs (including [DMBA][HSO_4_]) and basic ILs in terms of metal extraction from other types of biomass.^68,72^ Due to the limited degree of metal extraction achieved here using both ILs, further work should include investigating the metal extraction of these ILs at higher pretreatment severity or the use of [Hmim][Cl] for pretreatment of metal-contaminated duckweed at mild conditions.

#### 4.2.2 Saccharification

**Figure 9:**
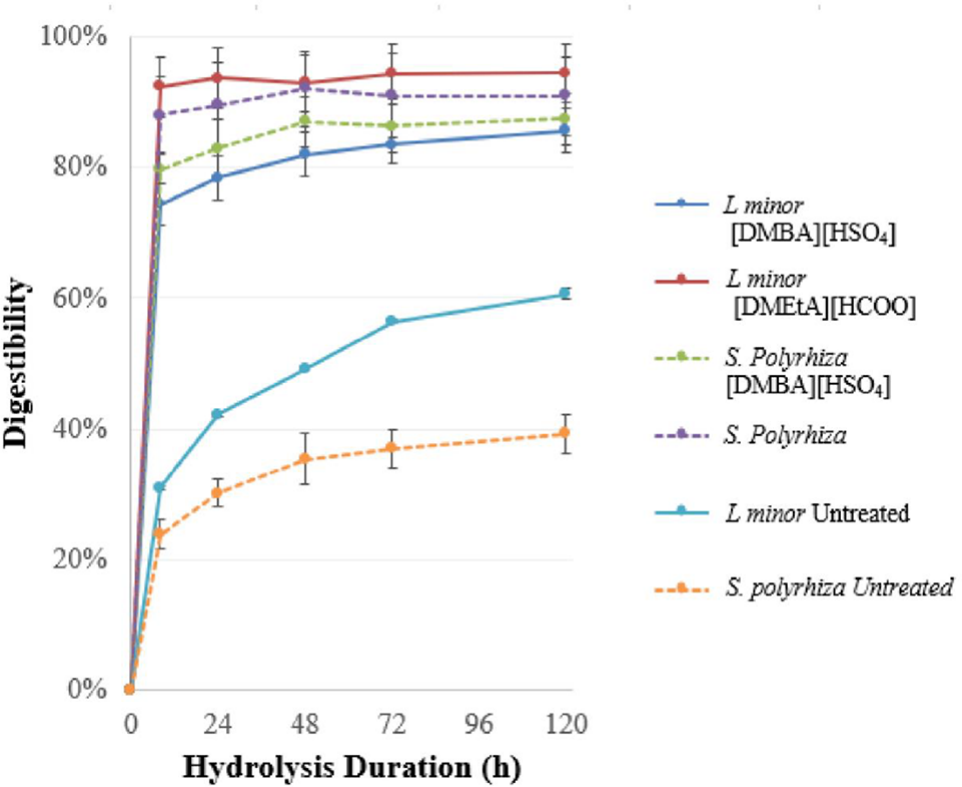
Saccharification kinetics of duckweed pulp pretreated using varying ionic liquids. All pretreatments were carried out with 20 wt % water and at a biomass loading of 10 wt %. Pretreatments using [DMEtA][HCOO] were carried out at 120 °C for 180 min, and pretreatments using [DMBA][HSO_4_] were carried out at 120 °C for 30 min. *Lemna minor* feedstocks were metal-contaminated, while *Spirodela polyrhiza* feedstocks were not.

The enzymatic hydrolysis curves for both conditions and the untreated feedstock are shown in Figure 9, with the corresponding curves using *S. polyrhiza* also included for reference. *L. minor* was found to be a highly digestible feedstock even when untreated, with a saccharification yield of 61% after 120 h. This is substantially higher than that of *S. polyrhiza* (39%) and appeared to still be increasing slowly after 120 h of hydrolysis. The reason for this may be this strain’s much higher proportion of starch relative to cellulose. The kinetics of the pretreated pulps were much faster than the untreated material, particularly using [DMEtA][HCOO]. After 8 h of hydrolysis, pulps pretreated using [DMEtA][HCOO] and [DMBA][HSO_4_] had reached 98% and 87% of their 120 h sugar yields. These are in line with the kinetics observed using *S. polyrhiza* under the same conditions (corresponding values of 97% and 91%).

Both conditions led to saccharification yields substantially higher than those of the untreated material, particularly at short hydrolysis durations. Using [DMEtA][HCOO], near-quantitative yields were obtained after 8 h of hydrolysis (92% for both), with yields not then increasing significantly. Compared to *S. polyrhiza*, pretreatments using this IL led to significantly higher yields at all points (10–11%). This is explained by the higher glucan recoveries using *L. minor* than *S. polyrhiza*.

Using [DMBA][HSO_4_] with *L. minor*, yields were much lower than those using [DMEtA][HCOO]. No such differences were observed between ILs in pretreating *S. polyrhiza*. The difference in this case may be due to the higher starch contents of *L. minor*, both in absolute terms (10% vs 3%) and as a proportion of glucan (49% vs 30%). As a result, the differences in starch recovery between both ILs lead to a larger difference in glucan composition of pulps, compared to those using *S. polyrhiza*. Pulps pretreated with [DMBA][HSO_4_] thus have a lower proportion of (more easily hydrolyzable) starch, making them less digestible as well as limiting their yields.

### 4.3 Mass Balance for IL Pretreatment of *S. polyrhiza*

**Figure 10:**
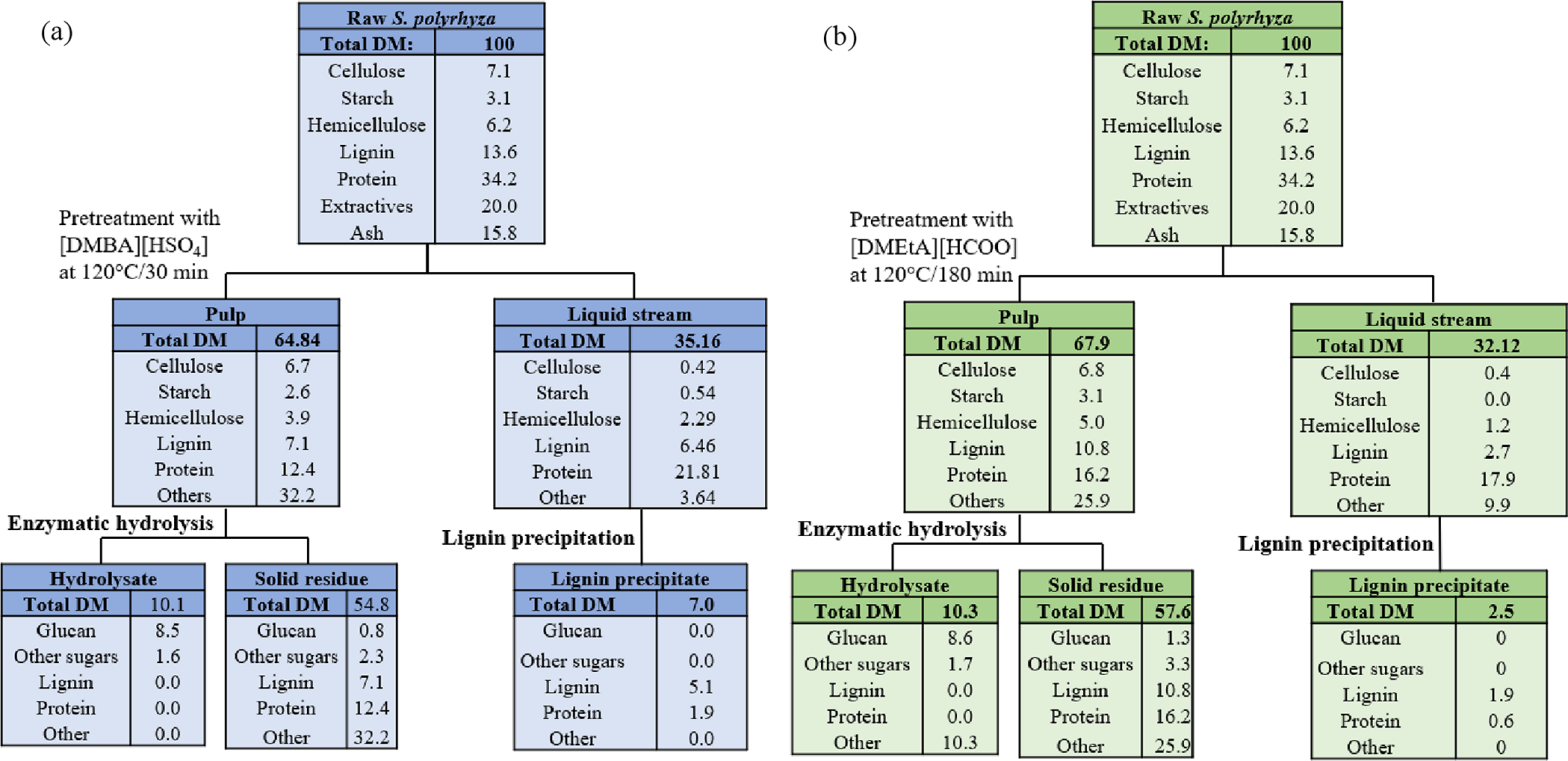
Mass balance for the PIL pretreatment of *S. polyrhyza* with (a) [DMBA][HSO_4_] at 120 °C and 30 min and (b) [DMEtA][HCOO] at 120 °C and 180 min. DM stands for dry matter. Other components include extractives, ash, and/or unaccounted products.

The mass balances for the pretreatment process with [DMBA][HSO_4_] and [DMEtA][HCOO] at their optimized conditions are shown in Figure 10a,b. It can be noticed that both PILs provided similar fractionation to the biomass with nearly 35% and 32% of the raw feedstock solubilizing into the IL phase, respectively. However, [DMBA][HSO_4_] solubilized more protein and hemicellulose due to its acidic nature. Nearly the same amount of sugars were produced upon saccharification of the pretreated materials, which is surprising given that the [DMEtA][HCOO] pulp contained slightly more hemicellulose. Additionally, lignin precipitation from [DMBA][HSO_4_] yielded more than double the amount from [DMEtA][HCOO], which shows one benefit of using the former PIL for lignin production. The overall low carbohydrate content of *S. Polyrhiza* may limit the efficiency of downstream ethanol production. Producing more valuable fermentation products such as organic acids like lactic or succinic or even butanol via ABE fermentation could offset such problem.

### 4.4 Sustainability of the IL Process

The duckweed biorefinery concept is focused on the bioremediation of wastewater while also producing different bio-based products such as proteins, lignin, and second-generation bioethanol. It is likely that the target product biorefinery would need to be tailored to the composition of input feedstocks due to their large potential variation in starch and protein content. Alternatively, a simple pre-processing stage (e.g., nutrient depravation of input duckweed) could be used to ensure sufficient starch content as in the work of Xu et al.^5^

Duckweed management for second-generation ethanol production represents a sustainable approach with the potential to advance the UN Sustainable Development Goals^103^ (SDGs) on multiple fronts. Duckweed is a fast-growing aquatic plant that can be cultivated on various water bodies without competing with food crops or depleting valuable arable land. This approach promotes SDG 15 (Life on Land) by reducing land degradation and SDG 6 (Clean Water and Sanitation) by utilizing wastewater for cultivation. Furthermore, this biomass efficiently captures CO_2_ from the atmosphere, contributing to SDG 13 (Climate Action). Ethanol production from duckweed provides an ecofriendly alternative to fossil fuels, thereby aligning with SDG 7 (Affordable and Clean Energy). Additionally, the potential for job creation in the cultivation and processing of duckweed can support SDG 8 (Decent Work and Economic Growth).

An IL-based duckweed biorefinery benefits from employing designer solvents that can be recovered and utilized, therefore reducing the environmental footprint of the pretreatment step. ILs allow for the efficient fractionation of the lignocellulosic components of duckweed, while also extracting contaminated heavy metals from the plants. The pretreated duckweed can be rapidly hydrolyzed into monosaccharides that can be then converted into 2G ethanol. A life cycle assessment is necessary to fully assess the overall sustainability of the process. However, there are a number of reviews in the literature that emphasize the growing importance of sustainable utilization of ILs.^104−106^

The use of protic ionic liquids derived from cheap bulk chemicals (in this case, formic acid, sulfuric acid, and simple alkylamines) with a simple one-step synthesis also increases the sustainability of this biorefinery process. In a recent lifecycle analysis by Baaqel et al., production of two alkylammonium hydrogen sulfate ionic liquids was found to have similar ecological impacts to glycerol and acetone production (measured across ecosystem quality damage, resources damage, and human health danger).^200^ Furthermore, while not carried out in this study, the easy post-pretreatment recovery (requiring only rotary evaporation of water to reduce water content) and successful reuse of protic alkylammonium hydrogen sulfate and alkylammonium carboxylate ionic liquids have been proven in several studies. When recycling [TEA][HSO_4_] (an alkylammonium hydrogen sulfate protic ionic liquid), Brandt-Talbot et al.^107^ reported 99% ionic liquid recovery over 4 cycles, with high pretreatment efficacy maintained throughout. Meanwhile, Nakasu et al.^108^ reported ionic liquid recoveries of 97% using [MEA][OAC] (an alkyammonium carboxylate protic ionic liquid) at 150 °C, with over 90% saccharification yield over six cycles through manipulation of the ionic liquid acid–base ratio to minimize acetamide formation. It is expected that [DMEtA][HCOO] would perform even better under recycling conditions due to the lower temperatures and inability of a single-carbon anion to degrade to an acetamide.

## 5 Conclusions

This study has provided a foundation for the IL pretreatment of duckweed. An ideal IL-based process for duckweed biorefinery would be able to take advantage of this feedstock’s three promising abilities: metal removal from dilute concentrations, starch accumulation, and protein accumulation. Such a process would, therefore, lead to recovery of a highly digestible pulp that preserves much of the starch and of a high-purity protein stream and would solubilize the extracted metals into the IL phase. This study has demonstrated that the first of these three goals is achievable under a range of conditions and was loosely optimized. Further studies should investigate the other two aims independently and then find a single operating point able to meet all three aims simultaneously.

## Supporting Information Available

Information regarding pretreatment data of the duckweed *Spirodela polyrrhiza* and *Lemna minor* at different times and temperatures; pictures describing the feedstock collection and appearance before and after pretreatment; an extended discussion of the ionic liquid pretreatment effect on pure starch and ^1^H-NMR and ^13^C-NMR spectra of the synthesized ionic liquids (PDF)
