# Supplementary material for: An Ionic Liquid-Based Biorefinery Approach for Duckweed Utilization
Source: ACS Sustain Resour Manag. 2024 Apr 30;1(5):842–56. doi: 10.1021/acssusresmgt.3c00008 (PMC11129354; doi:10.1021/acssusresmgt.3c00008)
Supplement: Supplementary file 1 — rm3c00008_si_001.pdf [file rm3c00008_si_001.pdf]

Electronic Supplementary Information

An Ionic liquid-based biorefinery approach for duckweed utilisation

Anton E. J. Firth[a], Pedro Y. S. Nakasu[a], Paul S. Fennell[a], Jason P. Hallett[a,b]

[a] Department of Chemical Engineering, Imperial College London, London, SW7 2AZ, UK.

[b] Corresponding Author.

Pretreated Duckweed Data

Pretreatment data for *Spirodela Polyrhiza* and *Lemna Minor* are provided in Table S1, with protein contents of pulp and lignin provided in Table S2. All pretreatments were performed at 150°C, at a solids loading of 10% wt., and 20% wt. water content.

Table S1. Duckweed Pretreatment Data.

Feedstock	Temp (°C)	Time (mins)	IL	Pulp Yield (%)	Lignin Yield (%)	Glucan Recovery (%)	Hemi. Removal (%)	Deligni-Fication (%)	Starch Content (%)	Cellulose Content (%)
Spirodela Polyrrhiza	90	60	[DMBA] [HSO_4]	67.6	36.7	94.2	59.5	30.4	5.0	9.8
		120		66.6	44.6	97.9	62.0	34.4	5.2	11.0
		180		63.9	50.3	87.19	68.5	40.9	4.0	10.4
	120	30		64.8	51.6	94.1	37.2	47.6	3.9	11.4
		60		56.9	61.3	73.0	51.9	54.9	1.8	11.8
		90		51.1	63.2	64.8	55.9	57.0	0.7	12.7
	150	15		60.7	58.2	75.1	77.5	46.5	2.4	10.7
		30		49.3	73.2	52.5	97.8	55.7	0.2	11.1
		45		45.9	80.7	51.5	100.0	64.1	0.1	11.8
	120	60	[DMEtA] [HCOO]	72.1	6.0	90.0	18.2	6.0	4.7	8.5
		180		67.9	18.4	95.1	20.2	19.2	5.0	10.1
		300		65.2	37.8	89.8	21.8	25.4	4.8	10.2

Lemna Minor	120	30	[DMEtA] [HCOO]	65.6	51.1	93.3	31.7	22.8	12.2	17.9
		180	[DMBA] [HSO_4]	70.0	31.6	98.9	7.8	-24.3	13.8	14.0

Table S2. Protein Content of Pulp and Lignin From Pretreated Duckweed.

Feedstock	Temp (ºC)	Time (mins)	IL	Pulp Protein Content (%)	Lignin Protein Content (%)
Spirodela Polyrhiza	90	60	[DMBA] [HSO_4]	26.9	16.7
		120		20.4	21.5
		180		15.8	25.0
	120	30		19.9	27.6
		60		12.3	29.9
		90		13.9	29.6
	150	15		16.8	29.7
		30		13.1	32.8
		45		9.6	29.4
	120	60	[DMEtA] [HCOO]	23.4	25.1
		180		24.9	25.3
		300		25.7	29.6
Lemna Minor	120	30	[DMBA] [HSO_4]	16.6	18.2
		180	[DMEtA] [HCOO]	18.8	15.4

Solid Recovered From Dialysis

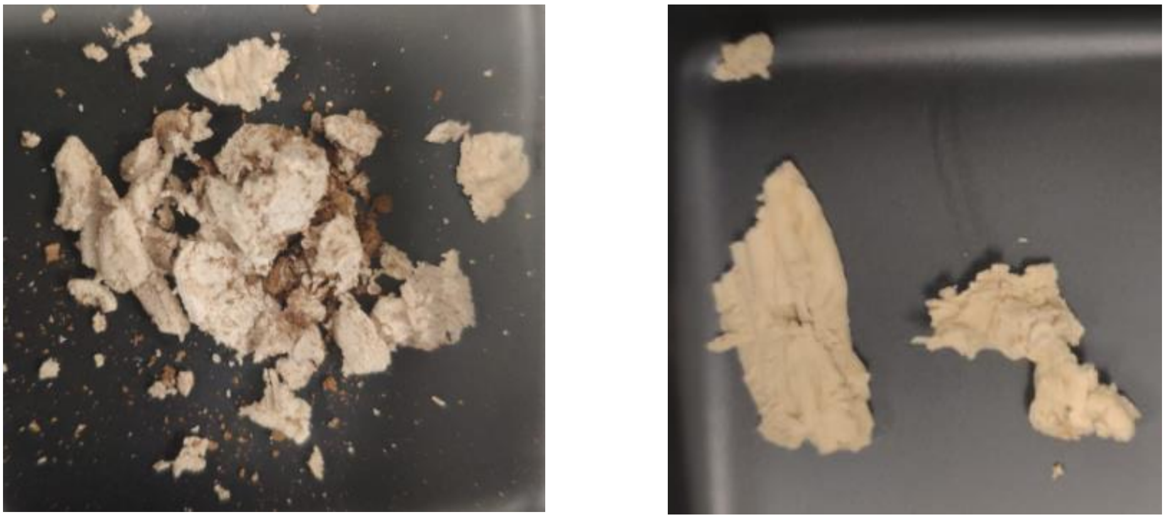

Figure S1. Solid recovered following dialysis of IL liquors. Figures are Right: from pretreatment with [DMBA][HSO_4] for 60 minutes at 120 °C, Left: from pretreatment with [DMEtA][HCOO] for 180 minutes at 120 °C. All pretreatments were carried out on *Spirodela polyrhiza*, with 20 wt% water, and at a biomass loading of 10% wt/wt.

Harvested Duckweed

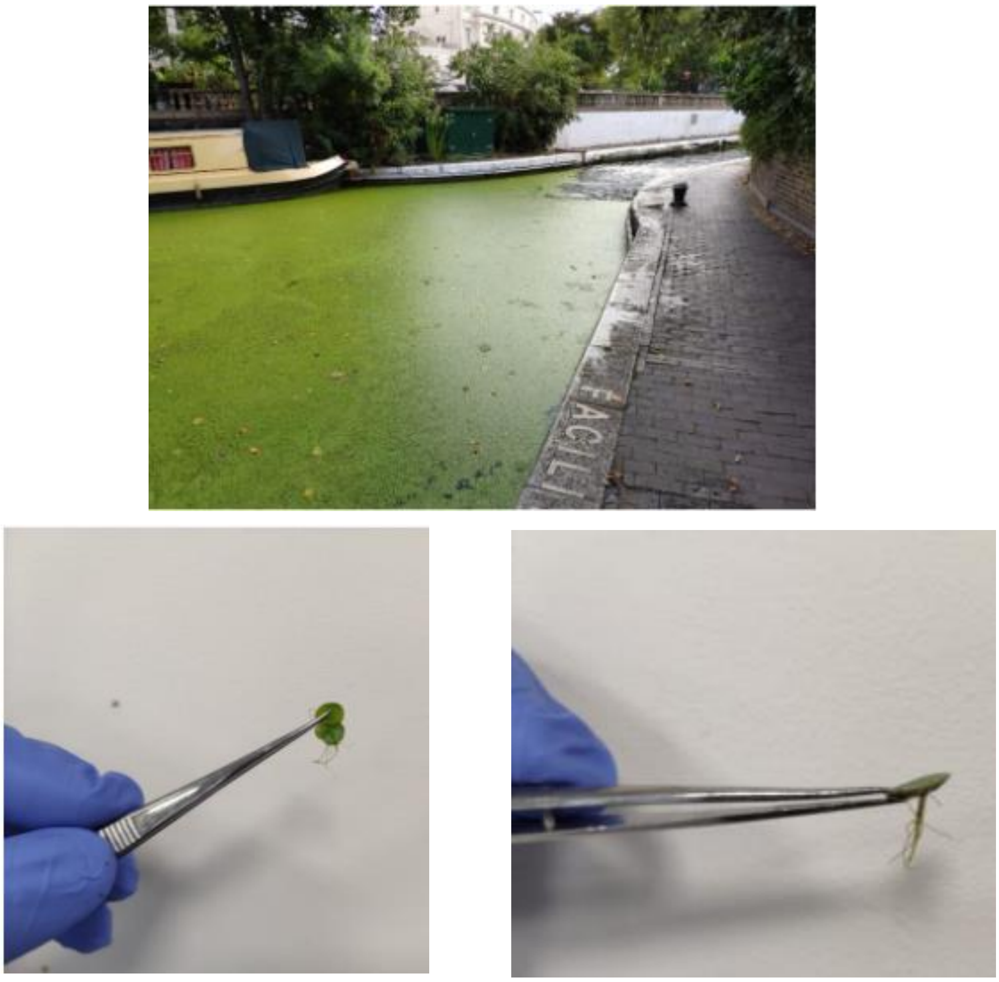

Figure S2. Harvested *Spirodela Polyrhiza*

Pretreatment of Pure Starch (extended discussion)

Four different types of starch were chosen in order to examine whether the effects of IL pretreatment were consistent across starches with different physical properties such as granule size, molecular weight, and amylose/amylopectin ratio. While these properties were not measured for the starches used, trends for these have been reported in the literature. Starch granule size has been shown to decrease in the order potato > wheat > corn > rice.[75,76] The overall molecular weight of several of the different starches has been reported to decrease in the order potato > wheat, corn.[76–78] The amylose/amylopectin trend in these studies decreased in the opposite order, explained by the lower degree of polymerisation of linear amylose compared to highly branched amylopectin. However in some reviews collating starch data, large, overlapping ranges of amylose/amylopectin are reported for the four types of starch used in this study.[75,79,80] It would, therefore, not appear possible to generalise the trends amongst the types of starches used.

[DMBA][HSO_4]

Results using [DMBA][HSO_4] are shown in Fig. 2a (main manuscript). None of the starches were found to dissolve in [DMBA][HSO_4] at either 90 ^oC or 120 ^oC, remaining as powders, and no significant precipitation was observed upon addition of ethanol. It can, therefore, be assumed that there was not total disruption of the starch granule structure. At 90 ^oC, no degradation was observed by HPLC, and recovered solids were quantitative for all four starches. This was somewhat surprising, considering the elevated temperature and highly acidic nature of this IL. Furthermore, the water-insoluble residue accounted for above 96% of input starch for all types except rice (89%). It would therefore appear as though starch remains fairly stable in [DMBA][HSO_4] at 90 ^oC, with slow rates of degradation, if any. Mass closures ranged from 100% to 104%, possibly indicating incomplete removal of IL from the starch from ethanol washing.

At 120 ^oC, degradation products were observed by HPLC (around two thirds of which were glucose, and one third was maltose). Such degradation products were found to account for 1-15% of input starch, increasing in the order potato (1%) < corn (3%) < wheat (11%) < rice (15%). At this elevated temperature, the ethanol-insoluble residue was found to increase substantially, while the water-insoluble residue decreased substantially. This is consistent with partial depolymerisation of the starch, forming oligomers that are ethanol-insoluble but water-soluble. The water-insoluble residue decreases in the order potato (37%) > corn (22%) > wheat (8%) > rice (4%), opposite to that of the degradation rates. Therefore, it would appear as though the starch stability during pretreatment decreases in the order potato > corn > wheat > rice. As the starches remained undissolved, and thus had their granular structure preserved, it is thought that the decreasing stability order in this acidic IL can be tied to a decreasing overall molecular weight, due in turn to a decreasing proportion of amylopectin.

There is also a significant unaccounted fraction across all four starches at 120 °C (22-51%). It is hypothesised that these are low molecular weight starch oligosaccharides that were soluble in the IL-ethanol mixture during the first ethanol wash. These oligosaccharides, which would not be detectable by HPLC, would have been discarded with the supernatant following this washing step. 120 °C is thus not a suitable temperature for starch preservation using [DMBA][HSO_4]. At this elevated temperature, the acidic nature of the IL leads to rapid starch depolymerisation. It is worth noting, however, that water-soluble, low-molecular weight starch oligomers have been targeted in previous studies due to their potential for modification.[20,81]

[DMEtA][HCOO] and [MEA][OAc]

Three of the starches were found to fully dissolve in these ILs, all except potato starch which partially dissolved. This is consistent with previous studies, showing that the $[HCOO]^-$ and $[OAc]^-$ anions are fairly effective at dissolving starch.[15,20,82–84] These basic anions break the granule structure of the powdered starch, leading to its dissolution, without extensive depolymerisation.[20] The resulting liquor was a thick gel. The starch was then precipitated upon addition of ethanol, forming a less viscous white-colourless gel that was insoluble in ethanol. Over the course of the ethanol washing procedure, this gel was replaced by a white solid. This was presumed to be an indication of the gel containing both starch and residual IL, with this residual IL being removed by ethanol washing and thus leaving only starch.[20] This fraction was recovered as a fairly hard, crumbly white solid for all starches.

Following the water wash, the appearance of the water-insoluble residue was dramatically different for all except potato starch, as shown in Fig. S3. Rice, wheat, and corn starch all produced a very hard, crystalline, white/colourless solid, whereas potato starch did not significantly change in appearance. This was observed at both 90 °C and 120 °C using both ILs. At 120 °C, the residue was completely colourless, whereas at 90 °C there were patches of white within the solid. The abundance of these patches decreased in the order corn > wheat > rice, seemingly correlated inversely with starch stability as determined using [DMBA][HSO_4]. CHN analysis was carried out on the water-insoluble residues, and no nitrogen (indicating presence of the cation) was detected, except 0.4 wt% in potato starch. It can therefore be concluded that the appearance of the recovered water-insoluble residues is not attributable to residual IL.

The changed appearance of the water-insoluble residue is thought to be due to starch retrogradation. This process describes the reassociation or recrystallization of the disrupted amylose and amylopectin chains in dissolved starch, which hydrogen bond together and form a crystalline structure.[85,86] Retrogradation is a complex process, dependent on a number of factors including time, water content, amylose/amylopectin ratio, and the molecular weights of the amylose and amylopectin fractions. [9,85–87] As a result, the change in appearance from the ethanol-insoluble residue to the water-insoluble residue

(i.e. by washing with water) is more challenging to explain using the available data. Hypotheses include removal of residual IL from the solid structure, removal of any non-retrograded starch sections, or that the ethanol-insoluble residue retrogrades into a more stable conformation in the water. However, determining the mechanisms responsible for this change are beyond the scope of this study, and should be examined further in future.

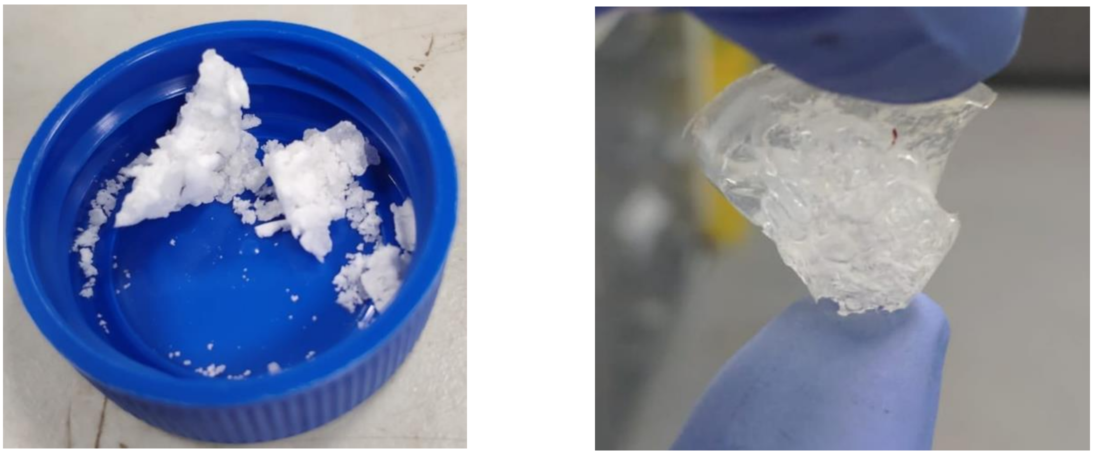

Figure S3. Water-insoluble residue of starches following pretreatment with [DMEtA][HCOO]. Potato starch is shown on the left, while rice starch is displayed on the right. All pretreatments were carried out at a solids loading of 10% wt/wt, with an IL water content of 20 wt%, at 120 °C for a duration of 30 minutes.

Results using [DMEtA][HCOO] and [MEA][OAc] are shown in Figs. 2c and 2d (main manuscript)., respectively. Potato starch displayed the lowest yields for the water-insoluble residue, despite appearing to be the most stable starch using [DMBA][HSO_4], and despite having similar ethanol-insoluble residue yields. It was also the only starch to be only partially solubilised, and not to substantially change in appearance following the water wash. This observation is also difficult to explain using the available data. The suggested theory is that the dissolved potato starch did not undergo similar, if any, retrogradation (for example due to its molecular weight or a lower amylose/amylopectin ratio). Amylose retrogrades more rapidly than amylopectin,[86–88] with the latter found to form at least two states: either integrating with the amylose or remaining as clusters of amylopectin.[88] These compounds are both insoluble in ethanol, but amylopectin is soluble in water.[89] The low quantities of water-insoluble residue could thus be due to the solubilisation of such free clusters of amylopectin.

The performance of both carboxylate ILs was very similar. At 90 °C, the water-insoluble residue was found to be near-quantitative for all starches, except potato starch. This was assumed to be due to the

low acidity of both ILs leading to little depolymerisation despite dissolution of the starch. At 120 °C, the water-insoluble residue was found to reduce slightly, with larger drops using [DMEtA][HCOO] except for potato starch. This would appear to suggest that [DMEtA][HCOO] is slightly more acidic than [MEA][OAc], which may be expected due to the former having a stronger constituent acid.[90] Conversely, potato starch displayed a lower water-insoluble residue in [MEA][OAc] despite it appearing to be less acidic. This may be due to differences in starch solubility and granule disruption between both ILs.

Over 100% mass balances were observed under all conditions. This was taken to be a sign of residual IL in the recovered solids, in the solid recovered following ethanol washing (due to higher solubility of these PILs in water than in ethanol, and due to the extra washing step). Similar results have been noted in the literature using similar ILs.[20] Additionally, some water may have been trapped within the retrograded, crystalline starch structure.

Both ILs would therefore appear to be promising solvents for pretreating starch-based biomass at elevated temperatures. However, for extended durations at 120 °C degradation may eventually increase to substantial levels.

[Hmim][Cl]

Results using [Hmim][Cl] are shown in Fig. 2b (main manuscript). Three of the starches were found to fully dissolve in this IL, all except potato starch which partially dissolved. This is consistent with literature reports, stating that the Cl^- anion is highly effective at dissolving starch,[8,14,20,25] but also describing incomplete disruption of potato starch granules in a similar IL.[14] At 90 °C, recovered starches had the same physical appearance as those pretreated with both neutral ILs. At 120 °C, however, almost no solid was recovered, except using potato starch.

At 90 °C, mass balances were all found to be above 100%, once again attributed to residual IL that was not removed by ethanol washing. [Hmim]Cl displayed the lowest water insoluble residues at both 90 °C and 120 °C, far lower than the similarly acidic [DMBA][HSO_4]. At 90 °C, despite quantitative ethanol-insoluble residues, water-insoluble residues were low (8-37%). These results indicate substantial depolymerisation of the starches in the acidic IL, forming ethanol-insoluble but water-soluble oligomers. The depolymerisation of starch dissolved in ILs containing the $[Cl]^-$ anion has been reported in several studies.[14,20,91] At 120 °C, only potato starch displayed any significant water-insoluble residue. The extent of starch loss in [Hmim][Cl] was assumed to be due to the dissolution of the starch in the IL, with the disruption of the granular structure allowing easier depolymerisation by this acidic IL; whereas all starches were insoluble in [DMBA][HSO_4] and thus did not depolymerise as

extensively. This would explain the lower water-insoluble residue at 90 ^{o}C. Furthermore, the higher concentration of dissolved starch in the solution may have led to more widespread but less complete degradation of starch. This would explain the lower proportion of starch degradation products detected using [Hmim][Cl] than using [DMBA][HSO_4] at 120 ^{o}C, and the higher proportion of unaccounted starch (attributed to ethanol-soluble starch oligomers).

Results using [Hmim][Cl] generally corroborate the starch stability order determined using [DMBA][HSO_4]. At both temperatures, potato starch displayed the highest water-insoluble residue. At 90 ^{o}C, wheat and corn starches showed similar water-insoluble residues, while at 120 ^{o}C, only corn starch showed ethanol-insoluble residue. Rice starch had the lowest water-insoluble residue at 90 ^{o}C. These observations are in line with the findings of Karkkainen et al, who found that amylopectin in potato starch degraded more slowly in [Bmim][Cl] than corn, wheat, and rice starches.[14] This therefore corroborates the previously proposed stability order of potato > corn > wheat > rice being due to decreasing amylopectin content.

[Hmim][Cl] is therefore not a suitable pretreatment solvent for starch-based biomass due to its acidity and ability to solubilise starches, leading to degradation and solubilisation of starch oligomers.

Ionic Liquid Synthesis - ^{1}H-NMR and ^{13}C-NMR

Proton and carbon NMRs for the synthesised ILs are provided in Figs. S4-11 all carried out in deuterated DMSO.

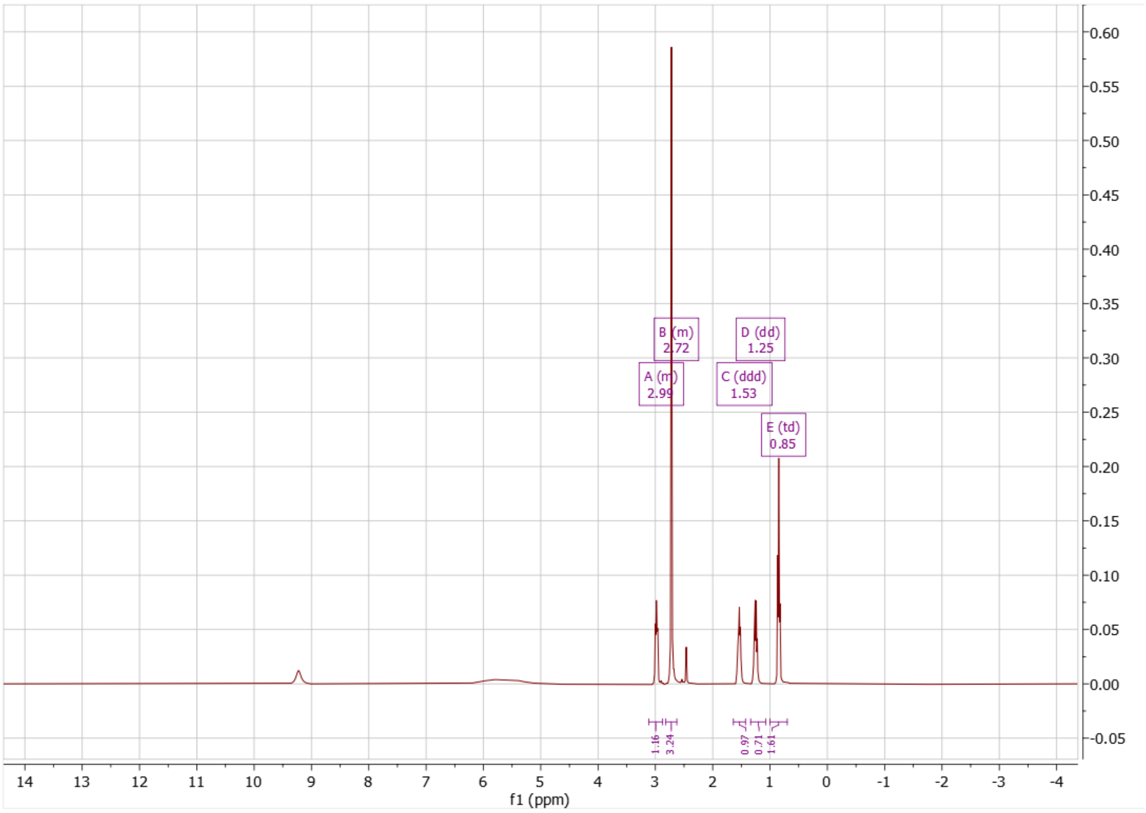

Figure S4. ^{1}H-NMR of [DMBA][HSO_4].

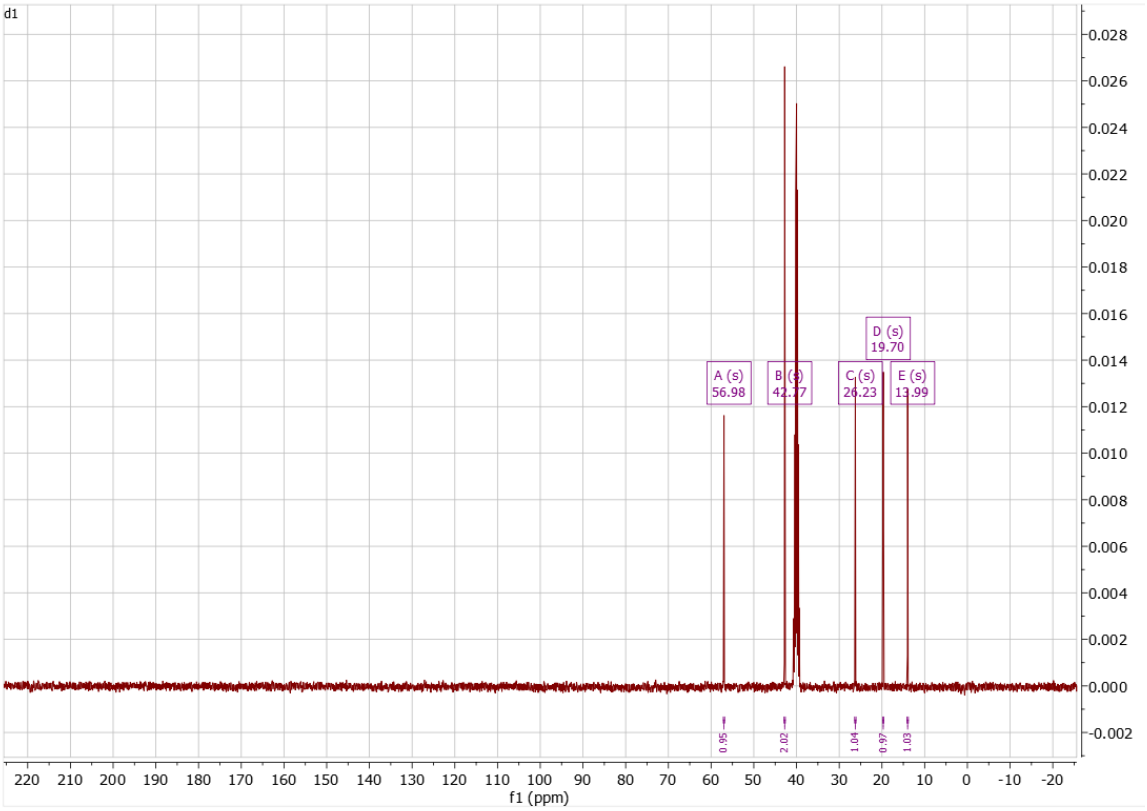

Figure S5. ^{13}C-NMR of [DMBA][HSO_4].

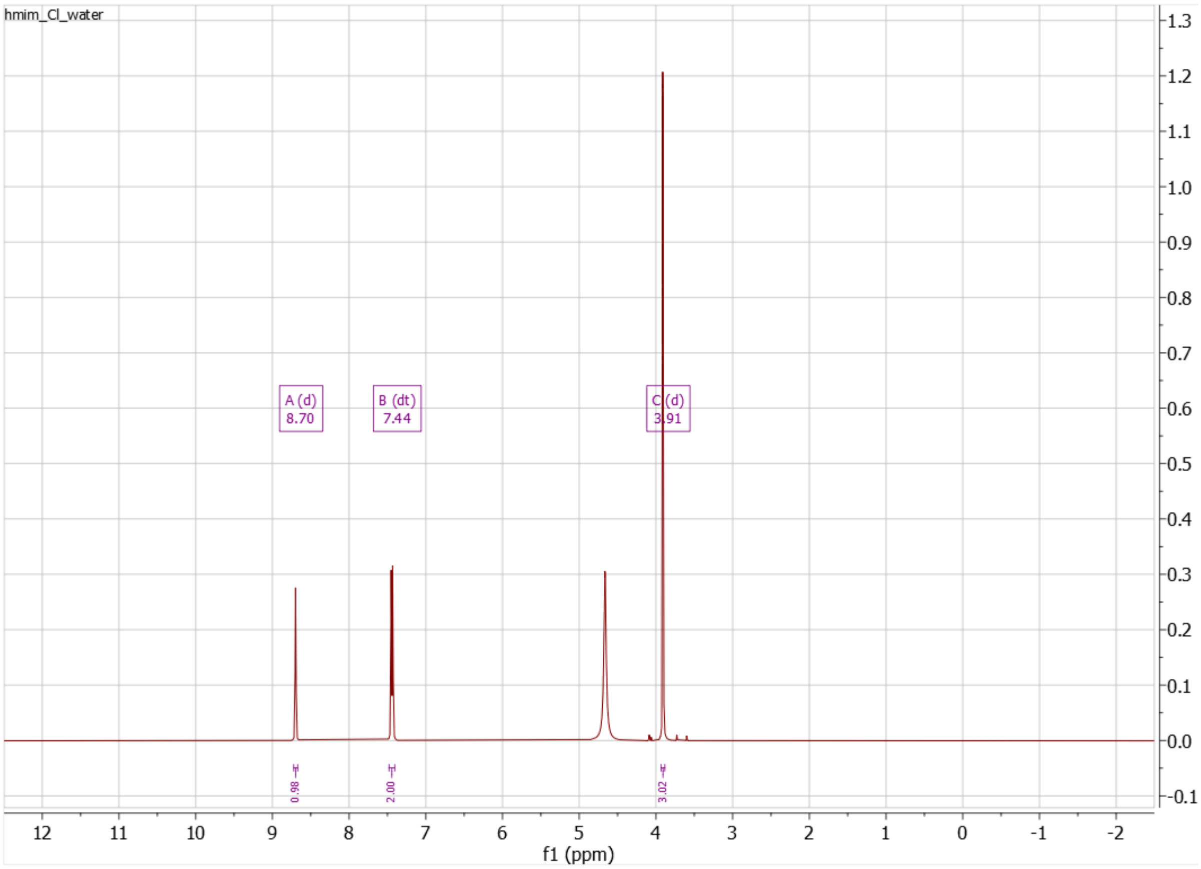

Figure S6. ^{1}H-NMR of [Hmim][Cl].

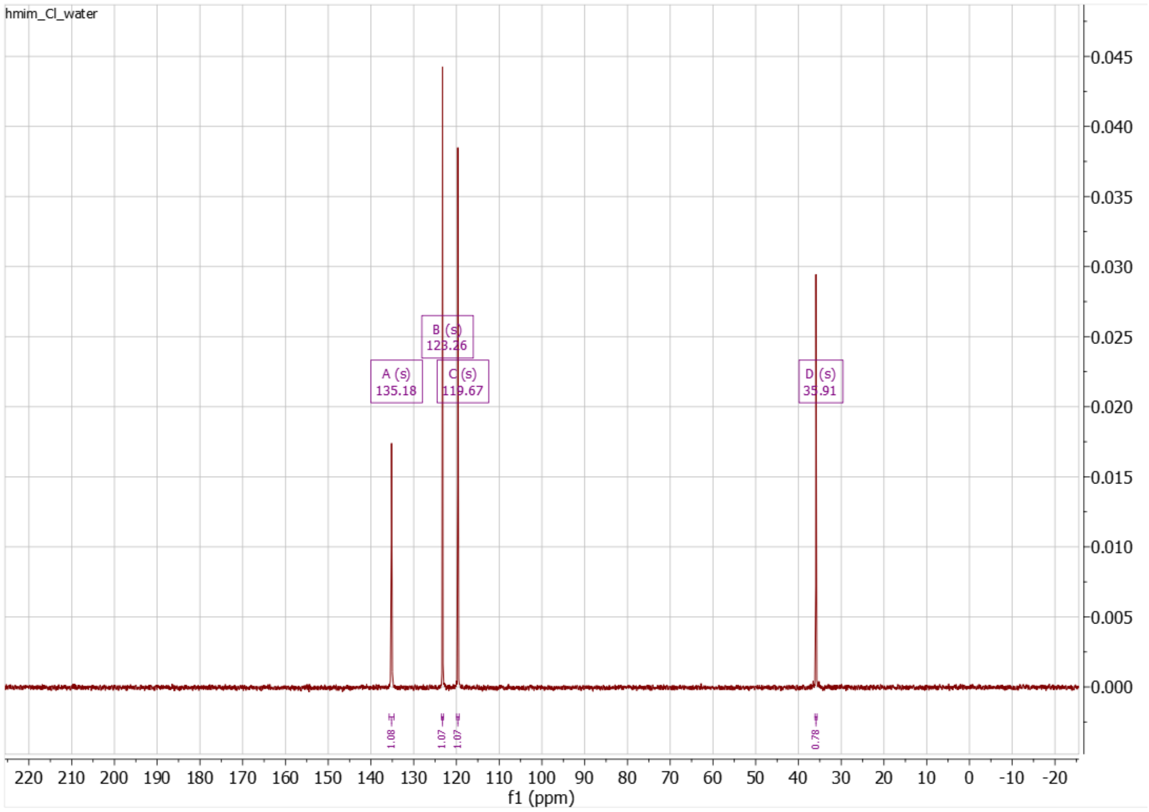

Figure S7. ^{13}C-NMR of [Hmim][Cl].

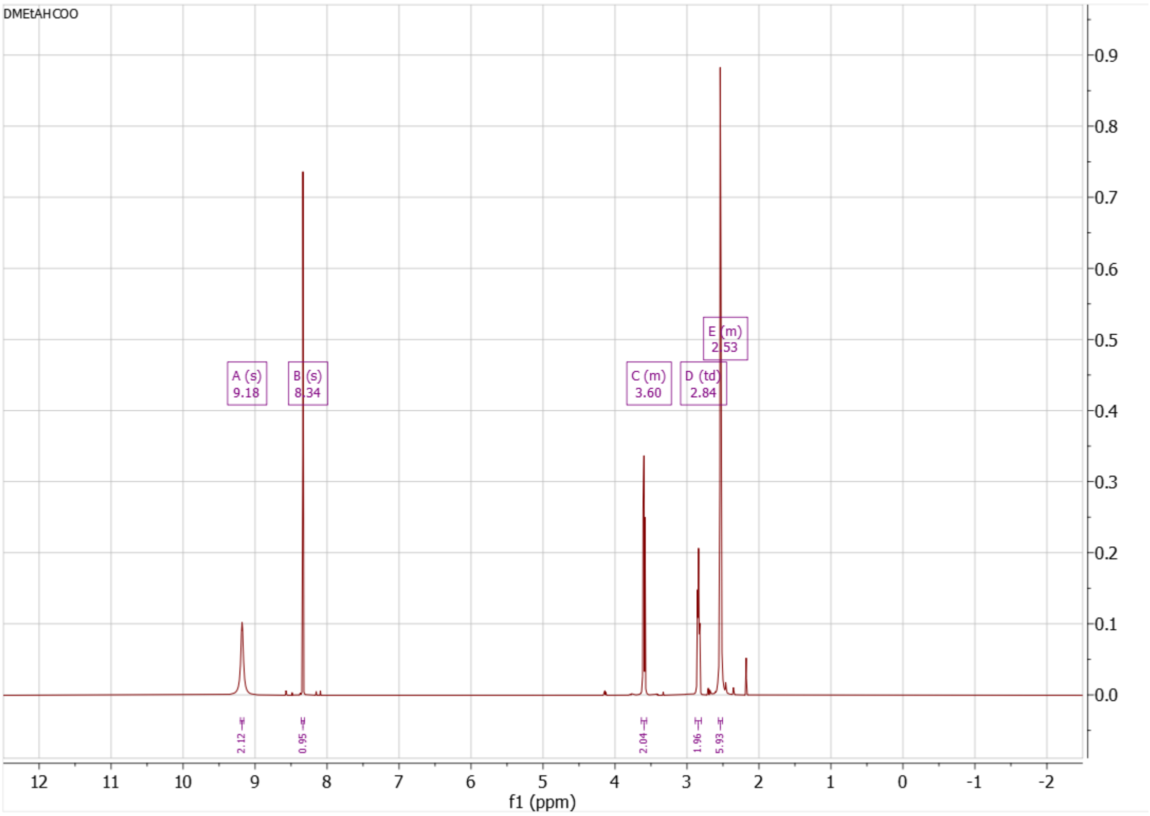

Figure S8. ^{1}H-NMR of [DMEtA][HCOO].

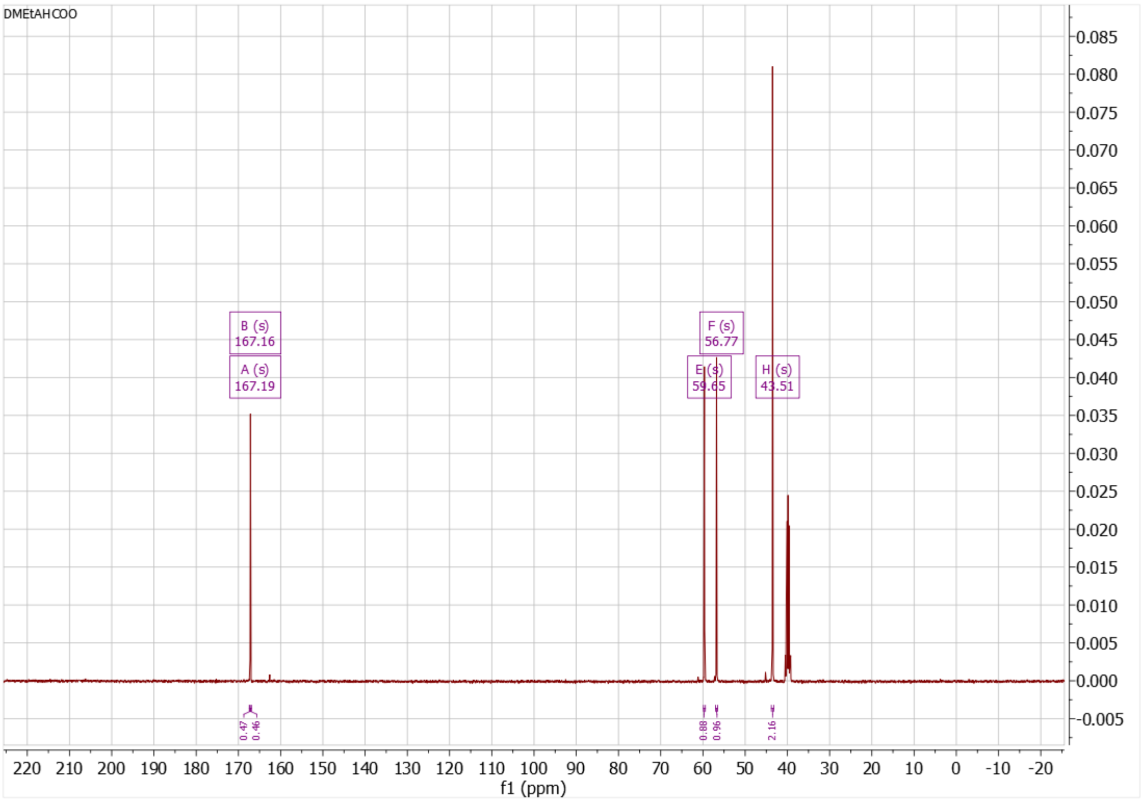

Figure S9. ^{13}C-NMR of [DMEtA][HCOO].

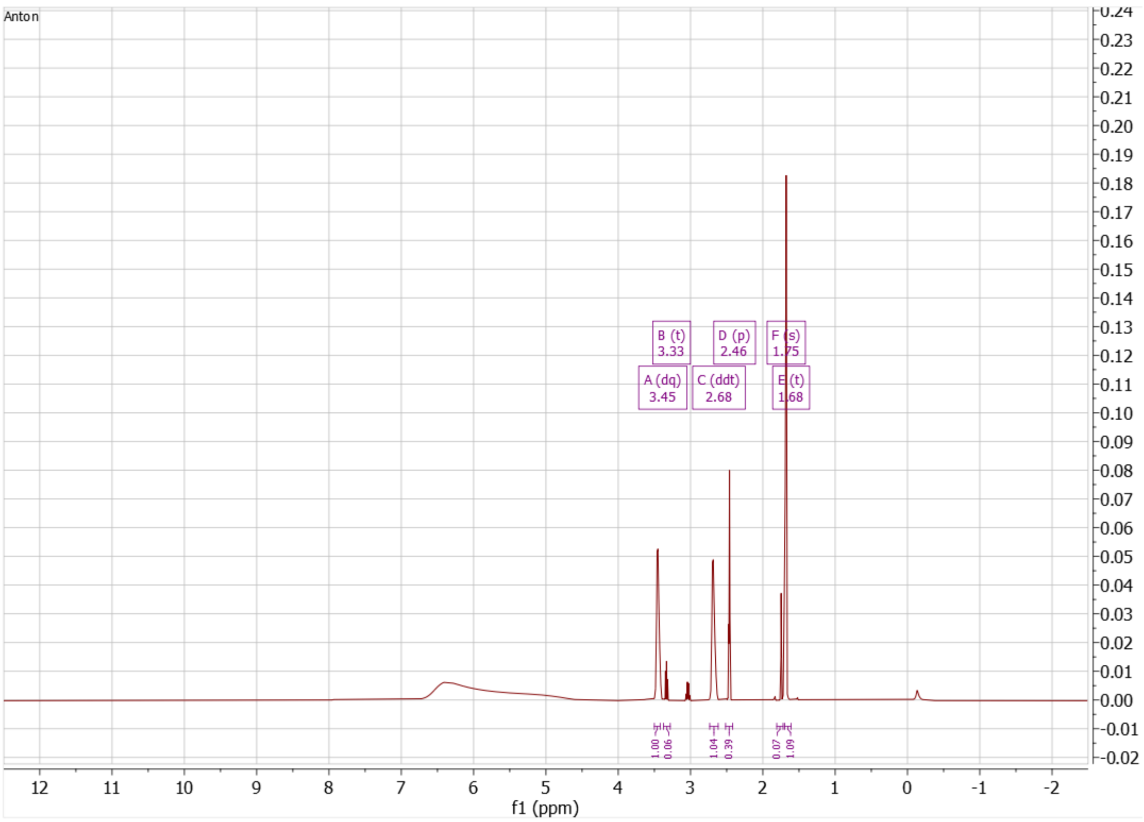

Figure S10. ^{1}H-NMR of [MEA][OAc].

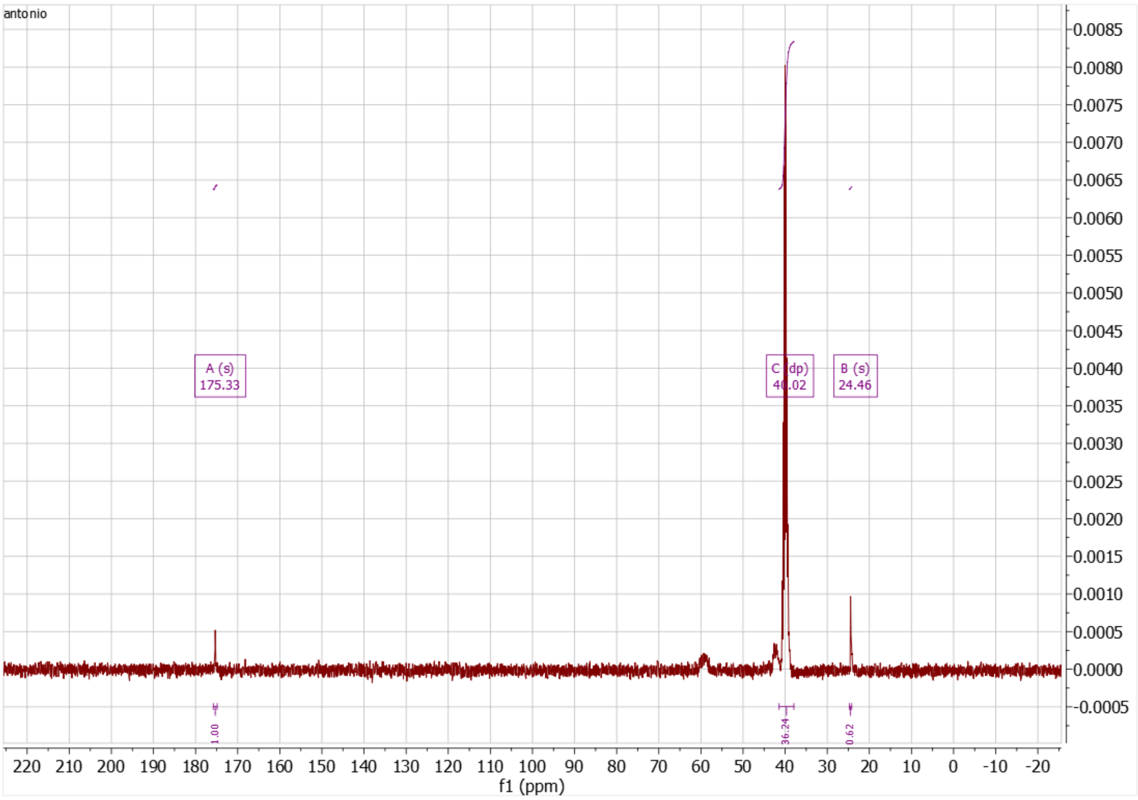

Figure S11. ^{13}C-NMR of of [MEA][OAc].
